# Disease progression in mice exposed to low-doses of aerosolized clinical isolates of *Burkholderia pseudomallei*

**DOI:** 10.1371/journal.pone.0208277

**Published:** 2018-11-30

**Authors:** Sylvia R. Trevino, Christopher P. Klimko, Matthew C. Reed, Michael J. Aponte-Cuadrado, Melissa Hunter, Jennifer L. Shoe, Joshua R. Meyer, Jennifer L. Dankmeyer, Sergei S. Biryukov, Avery V. Quirk, Kristen A. Fritts, Steven J. Kern, David P. Fetterer, Lara J. Kohler, Ronald G. Toothman, Joel A. Bozue, Christopher W. Schellhase, Norman Kreiselmeier, Sharon P. Daye, Susan L. Welkos, Carl Soffler, Patricia L. Worsham, David M. Waag, Kei Amemiya, Christopher K. Cote

**Affiliations:** 1 Bacteriology Division, United States Army Medical Research Institute of Infectious Diseases (USAMRIID), Fort Detrick, Frederick, MD, United States of America; 2 Pathology Division, United States Army Medical Research Institute of Infectious Diseases (USAMRIID), Fort Detrick, Frederick, MD, United States of America; 3 BioStatisitics Division, United States Army Medical Research Institute of Infectious Diseases (USAMRIID), Fort Detrick, Frederick, MD, United States of America; Tulane University School of Medicine, UNITED STATES

## Abstract

Mouse models have been essential to generate supporting data for the research of infectious diseases. *Burkholderia pseudomallei*, the etiological agent of melioidosis, has been studied using mouse models to investigate pathogenesis and efficacy of novel medical countermeasures to include both vaccines and therapeutics. Previous characterization of mouse models of melioidosis have demonstrated that BALB/c mice present with an acute infection, whereas C57BL/6 mice have shown a tendency to be more resistant to infection and may model chronic disease. In this study, either BALB/c or C57BL/6 mice were exposed to aerosolized human clinical isolates of *B*. *pseudomallei*. The bacterial strains included HBPUB10134a (virulent isolate from Thailand), MSHR5855 (virulent isolate from Australia), and 1106a (relatively attenuated isolate from Thailand). The LD_50_ values were calculated and serial sample collections were performed in order to examine the bacterial burdens in tissues, histopathological features of disease, and the immune response mounted by the mice after exposure to aerosolized *B*. *pseudomallei*. These data will be important when utilizing these models for testing novel medical countermeasures. Additionally, by comparing highly virulent strains with attenuated isolates, we hope to better understand the complex disease pathogenesis associated with this bacterium.

## Introduction

*Burkholderia pseudomallei* is the gram negative bacterium that causes melioidosis. It exists in soil and water and is endemic in Southeast Asia and northern Australia [1]. More recently, cases of the disease have been seen in other tropical regions including those found in the western hemisphere [2–7]. Melioidosis occurs primarily in humans who have been in contact with contaminated water or soil [8], however inhalation of aerosolized bacteria is also known to be a route of exposure in areas where monsoonal rains, flooding, and high winds occur [9–11].

The incubation period of melioidosis in exposed individuals can range from days to years after primary exposure to the bacterium, and it can persist for decades without clinical signs or symptoms [8, 12, 13]. The long incubation period may be due to the intracellular life cycle of the pathogen, allowing it to avoid detection by the host immune system [14]. There are no truly distinctive features of melioidosis. Acute cases of melioidosis are characterized by generalized signs and symptoms, including fever, malaise, pneumonia, and sepsis. Abscess formation can be widespread, but commonly occurs in the liver, spleen, skeletal muscle, prostate, and kidney [8]. Melioidosis may also present as a chronic disease characterized by signs and symptoms that may persist for years, as reviewed by Brett, DeShazer, and Vietri [8]. The symptoms of the chronic form are usually milder than those of the acute form, further challenging diagnoses. Persons exposed to *B*. *pseudomallei* with high risk factors such as diabetes, renal failure, and alcoholism are more likely to develop melioidosis [8, 10, 15].

The mechanisms of pathogenicity for *B*. *pseudomallei* are not well characterized particularly when the disease is acquired by aerosol exposure. *B*. *pseudomallei* is designated as a Tier 1 agent due to its potential use as a biological threat agent [8]. Exposure to aerosolized bacteria is a primary concern when developing novel medical countermeasures and therefore is an important route for evaluating our panel of 11 strains of *B*. *pseudomallei* in context of biodefense. This panel includes clinical isolates and commonly used laboratory strains of *B*. *pseudomallei* [16]. We have previously examined this collection of isolates using the intraperitoneal model of infection and other in vitro analyses [17]. In this report we measure virulence by median lethal dose (LD_50_) determinations using the inhalational murine models of infection. Furthermore, we selected three *B*. *pseudomallei* strains that represented the most virulent and least virulent strain(s) to carry out a comparative serial sample study to obtain further information on the pathogenicity of these widely divergent strains. We report the bacterial burden in blood and tissues, histopathology observed during the disease course, and immune responses mounted during the infection in these models. These data will further contribute to existing knowledge of the aerosol murine model of melioidosis helping to understand and characterize animal models of disease.

## Materials and methods

### Animal challenges

Groups of BALB/c or C57BL/6 mice (Charles River-Frederick, MD; female 7–10 weeks of age at time of exposure to bacteria) were exposed to aerosolized bacteria from low passage and well-defined stocks of *B*. *pseudomallei* [16, 17]. The bacteria used were grown in 4% glycerol (Sigma Aldrich, St. Louis, MO)-1% tryptone (Difco, Becton Dickinson, Sparks, MD) and 5% NaCl (Sigma Aldrich, St. Louis, MO) broth (GTB) at 37°C with shaking at 200 rpm and were harvested from a late log phase culture. The bacteria were resuspended in GTB and quantified via OD_620_ estimations. The actual delivered doses of bacteria were then verified by plate counts on sheep’s blood agar (Trypticase soy agar with sheep blood-SBA) plates (Remel^TM^, ThermoFisher Scientific, Waltham, MA). Exposure to aerosolized bacteria was accomplished as previously described [18–20]. Mice were transferred to wire mesh cages and wire mesh cages were placed in a whole-body aerosol chamber within a class three biological safety cabinet located inside the BSL-3 laboratory. Mice were exposed to aerosolized *B*. *pseudomallei* isolates on separate days. Aerosols created by a three-jet collision nebulizer for 10 min at a constant flow rate of 19 L/min followed by a five minute wash cycle. Following the wash cycle, mice were removed from the aerosol chamber and transported back to their housing room. The aerosolization was performed at ambient temperature and humidity. The generated aerosol was sampled with an all-glass impinger (AGI) sampling at a rate of 6 L/min. AGI samples were analyzed by plating on SBA plates. CFU calculations were done to determine the inhaled dose of *B*. *pseudomallei*. The dose was calculated using the following formula: Dose = [Aerosol] (μg/mL) × minute volume (mL) × exposure time (min). Minute volume of mice was estimated using the mean weight of all mice on the day of exposure and Guyton’s Formula [21].

For dissemination studies following aerosol challenge, mice were euthanized by exsanguination under deep anesthesia (intraperitoneal injection of approximately 200 μl of a solution containing 6.7 mg/ml ketamine, 0.1 mg/ml acepromazine, and 0.7 mg/ml xylazine) on days 1, 2, 3, 7, 9/10, 14, 21, 28 and 59/60 post-infection and lungs and spleen samples were examined for both bacterial burden and immune response to the ensuing infection. Tissues were harvested, weighed, homogenized, and then CFU were enumerated on SBA plates. The limit of detection for spleen and lungs was approximately 5 CFU/ml. Due to blood volume constraints, the limit of detection for blood was approximately 50 CFU/ml. Confirmatory bacterial identification was also performed using *Burkholderia cepacia* selective agar plates (Remel^TM^, ThermoFisher Scientific, Waltham, MA).

### Ethics statement

Animal research at the United States Army Medical Research Institute of Infectious Diseases (USAMRIID) was conducted under an animal use protocol approved by the USAMRIID Institutional Animal Care and Use Committee (IACUC) in compliance with the Animal Welfare Act, PHS Policy, and other Federal statutes and regulations relating to animals and experiments involving animals. The facility where this research was conducted is accredited by the Association for Assessment and Accreditation of Laboratory Animal Care International (AAALACi) and adheres to principles stated in the Guide for the Care and Use of Laboratory Animals (National Research Council, 2011). Challenged mice were observed at least daily for up to 60 days for clinical signs of illness. Early interventions endpoints were used during all studies and mice were humanely euthanized when moribund, according to an endpoint score sheet. Animals were scored on a scale of 0–11: 0–2 = no significant clinical signs (e.g., slightly ruffled fur); 3–7 = significant clinical symptoms such as subdued behavior, hunched appearance, absence of grooming, hind limb issues of varying severity and/or pyogranulomatous swelling of varying severity (increased monitoring was warranted); 8–11 = distress. Those animals receiving a score of 8–11 were euthanized. However, even with multiple observations per day, some animals succumbed to the infection prior to meeting early endpoint euthanasia criteria.

### Spleen cell preparation

Splenocytes were prepared essentially as previously described [18]. Briefly, spleens were excised from mice (N = 5 mice for most time points), weighed, and disaggregated in RPMI 1640 medium (Life Technology, Grand Island, NY) containing 25 mM HEPES, 2 mM glutamine (wash medium) to make the spleen extract. Aliquots of the spleen homogenate were saved for cytokine/chemokine determination and stored at -70° C. Samples were irradiated with approximately 2.1 kGy of gamma-radiation and confirmed sterile by testing 10% of the sample before use. CFU in non-irradiated aliquots of the homogenate were determined on SBA with undiluted extract or 10-fold dilutions in sterile GTB. Plates were incubated at 37° C for two-three days before counting CFU. Red cells in the spleen homogenate were lysed with ACK (Ammonium-Chloride-Potassium) Lysing Buffer (BioWhittaker, Walkersville, MD) after the extract was diluted with wash medium and cells pelleted by centrifugation at 1,200 rpm for 10 min. Splenocytes were then washed once and suspended in complete medium [wash medium containing 10% heat-inactivated fetal calf serum (Life Technology), 1 mM sodium pyruvate, 0.1 mM non-essential amino acids, 100 U/ml of penicillin, 100 μg/ml streptomycin, and 50 μM 2-mercaptoethanol] and cells counted.

### Lung homogenate preparation

Lungs were removed and homogenized in RPMI medium using disposable precision homogenizers (Covidien, Dublin, Republic of Ireland). Aliquots of the lung homogenate were saved for cytokine/chemokine determination and stored at -70° C. Samples were irradiated with approximately 2.1 kGy of gamma-radiation and confirmed sterile by testing 10% of the sample before use. CFU in non-irradiated aliquots of the homogenate were determined on SBA with undiluted extract or 10-fold dilutions in sterile GTB. Plates were incubated at 37° C for two-three days before counting CFU.

### Splenocyte composition

Splenocyte cell composition was determined essentially as previously described [18]. Approximately 1x10^7^ splenocytes from each mouse were washed in FACS staining buffer (FSB) (1XPBS, 3% fetal calf serum, Life Technologies), and fixed in FSB containing 4% formaldehyde (Pierce, Rockford, IL) at 4°C. The cells were washed in FSB and then distributed into a microtiter plate (5x10^5^ cells/well), and nonspecific binding was inhibited by the addition of Fc Block (BD Biosciences, San Jose, CA). Cells were labeled with the following specific antibodies (BD Biosciences): CD4 T cells, CD4-PE/CD44-FITC; CD8+ T cells, CD8-PE/CD44-FITC; B cells, B220-PE/CD86-FITC; monocytes/macrophages, CD11b-PE/CD44-FITC; NK cells, CD49b-PE/CD44-FITC; and granulocytes, Ly6G-PE/CD44-FITC. Corresponding isotype controls were used and all were incubated for 60 min on ice. All samples were fixed in FSB with 4% formaldehyde and stored at 4°C until analysis. Cells were identified with a BD FACSCalibur using CellQuestPro software (BD Biosciences). Splenocytes from uninfected BALB/c mice were prepared as described above and used as normal, uninfected controls.

### Antibody ELISAs

Immunoglobulin (Ig) class IgG titers in challenged mice were determined by an ELISA performed in 96-well, Immulon 2 HB, round-bottom plates (ThermoFisher). Irradiated *B*. *pseudomallei* K96243 cells used as standard antigens for comparison, were diluted in 0.1 M carbonate buffer, pH 9.5, to a concentration of 10 μg/ml, and 50 μl of diluted cells were placed into wells. Plates were stored overnight at 4°C. The plates were washed with washing solution (1× PBS, 0.05% Tween 20), and incubated with 100 μl of blocking solution (1× PBS, 1% Casein) for 30 min at 37°C. Twofold dilutions of mouse sera were made with antibody assay diluent (1X PBS, 0.25% Casein) in triplicate, and plates were incubated for 1 h at 37°C. After the plates were washed, 50 μl of 1/5,000-diluted anti-IgG-horseradish peroxidase conjugate (Southern Biotechnology Associates, Inc., Birmingham, Ala.) was added to each well, and plates were incubated for 30 min at 37°C. After the plates were washed, 50 μl of a buffered hydrogen peroxide and 3,3′,5,5′-tetramethylbenzidine solution (Pierce, ThermoFisher) was added to each well, and plates were incubated for 20 min at 37°C. The reaction was stopped with 25 μl of 2 N sulfuric acid, and the amount of bound antibody was determined colorimetrically by reading at 450 nm with a reference filter (570 nm). The antibody titer results are reported as the reciprocal of the highest dilution giving a mean OD of at least 0.1 (which was at least twice the background) ± 1 SD.

### Cytokine/Chemokine expression

Cytokines/chemokines expression levels in mouse sera, spleen homogenates, and lung homogenates (N ≥ 5 for all time points) were measured by Luminex Mag Pix (Life Technology, Grand Island, NY) as per manufacturer directions. Spleen homogenates and sera from uninfected mice were used as normal, uninfected controls (N ≥ 4 BALB/c; N = 5 C57BL/6). The levels (pg/ml) of the following 20 cytokines/chemokines were measured: FGF-β, GM-CSF, IFN-γ, IL-1α, IL-1β, IL-2, IL-4, IL-5, IL-6, IL-10, IL-12 (p40/p70), IL-13, IL-17, IP-10, KC, MCP-1, MIG, MIP-1α, TNF-α, and VEGF. Heat maps were generated to report the fold-increase [test results (pg/ml)/ naïve results (pg/ml)] in cytokine or chemokine levels after exposure (in days) of mice to HBPUB10134a, MSHR5855, or 1106a.

### Histological pathology

Postmortem tissues were collected from representative euthanized mice and fixed in 10% neutral buffered formalin for ≥ 21 days. Samples were embedded in paraffin and sectioned for hematoxylin and eosin (HE) staining, as previously described [18, 22]. In order to identify bacterial antigen, immunohistochemistry was performed on selected formalin fixed tissues as previously described using a rabbit polyclonal antibody for *Burkholderia* spp. exopolysaccharide [23].

### Statistical analyses

The LD_50_ estimates represent the concentration for which experimenters may expect survival rates of 50% when repeating these assays under the methods and conditions described in this report, even though such a dose may not be achievable in practice, i.e. there may be no experimentally feasible dose (< 1 CFU) that would be expected to produce 50% lethality.

Estimation of the LD_50_ is made by Probit analysis with Bayesian estimation. The following probit model was fit: Φ^-1^(*p*_i_) = α_i_ + *β*_i_ ·Dose; where i indexes the strain or preparation, and α and *β* are the intercept and slope, respectively, for each combination of preparation and mouse strain. The priors for this model are αi ~Cauchy (0; 10) and β_i_ ~ Cauchy (0; 10). Samples were drawn from the posteriors using Hamiltonian Monte Carlo as implemented in Stan using four chains each with a warmup of 2,500 draws followed by 12,500 samples for a total of 50,000 posterior points. All Bayesian estimates are presented with 95% highest posterior density (HPD) intervals. Dosing is presented in the log10 transformation. This model allows for the direct comparisons of relative challenge potencies by comparing the posterior distributions of the range of lethal doses for each pair of strains compared. All Bayesian analyses were performed using Stan 2.1.0. All other statistics were performed using R 3.1.1. When comparing dose curves between strains of mice, all dose response curves were assumed to have common slope on the Log dose scale. Analysis is implemented in SAS proc Probit and SAS Proc Genmod, SAS version 9.4. No adjustment is applied for multiple comparisons. Immunological data were compared by t-test, with results summarized as the geometric mean, or the ratio of geometric means to naïve control.

## Results

### LD_50_ determinations in mice after exposure to aerosolized *B*. *pseudomallei* clinical isolates

**Fig 1:**
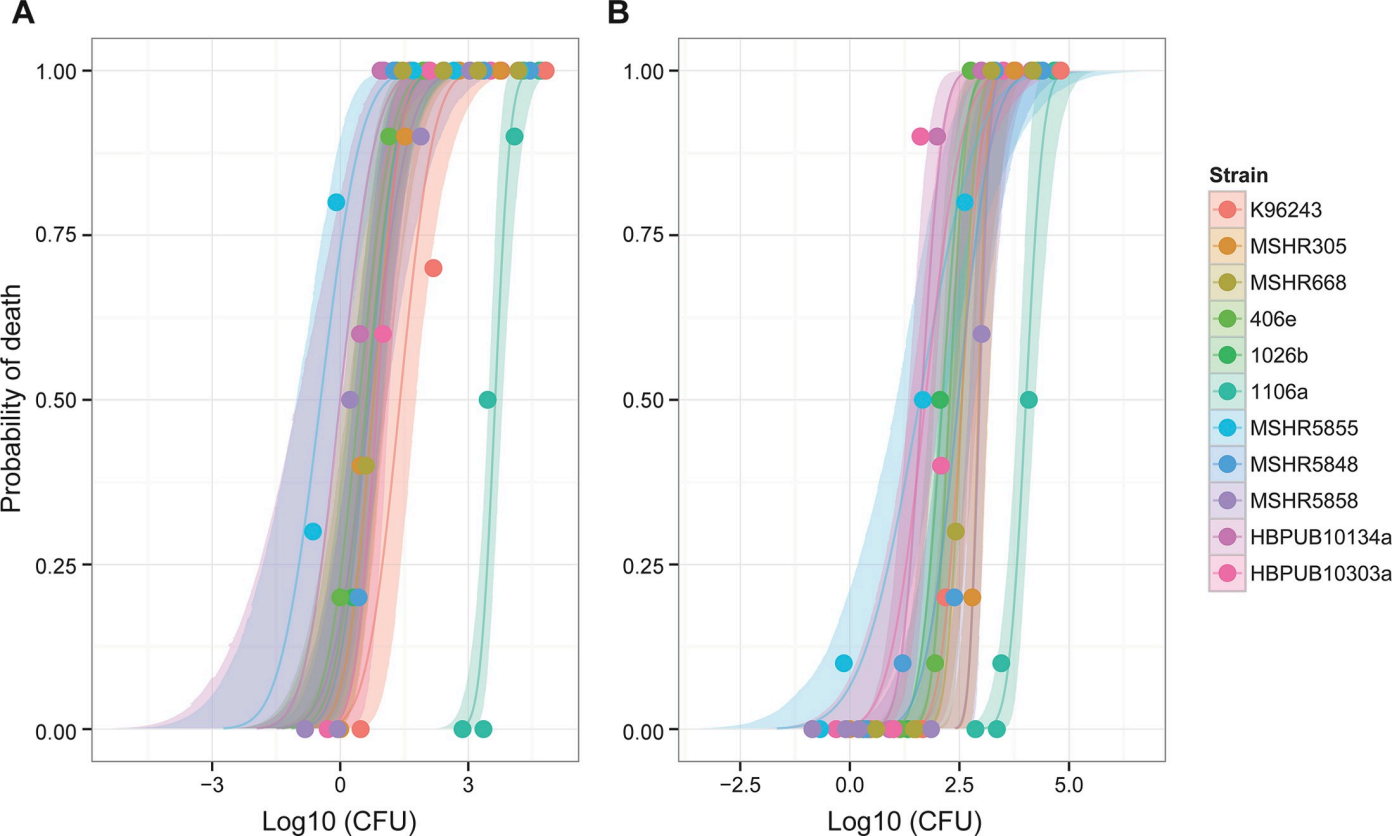
Plot of dose response curves for each *B*. *pseudomallei* clinical isolate in **(A)** BALB/c mice and **(B)** C57BL/6 mice.

As described previously a panel of *B*. *pseudomallei* clinical isolates was assembled in conjunction with the U.S. Department of Health and Human Services Biomedical Advanced Research and Development Authority (BARDA) and the U.S. Department of Defense Threat reduction Agency (DTRA) [16, 17]. Importantly, these strains were considered low passage and well characterized human clinical isolates. Groups of 10 mice were exposed to escalating doses of each isolate of *B*. *pseudomallei* in order to determine LD_50_ values in both BALB/c and C57BL/6 mice. As shown in **Table 1**, LD_50_ values were determined for each aerosolized *B*. *pseudomallei* strain in the panel for both BALB/c and C57BL/6 mice and the 95% credible intervals are also included. As depicted in **Fig 1**, isolate 1106a was the most apparent outlier regardless of mouse strain evaluated. The same observations were reported when mice were infected via intraperitoneal injection [17]. These data were then used to down select a subset of *B*. *pseudomallei* strains that included the most virulent and least virulent *B*. *pseudomallei* strains for further studies. We previously reported on K96243 using a low passage isolate of this common laboratory strain [18]. We determined that we would continue to characterize and assess HBPUB10134a and MSHR5855, the most virulent clinical isolates from Thailand and Australia, respectively, in this strain panel. Additionally, we continued to characterize strain 1106a because of the apparent attenuation of virulence in this clinical isolate could be of value for future pathogenesis studies or subsequent development of novel medical countermeasures.

**Table 1:** LD_50_ estimates and 95% HPD credible intervals of lethal doses for aerosolized *B*. *pseudomallei* clinical isolates.

	BALB/c				C57BL/6	
		95% HPD Credible Interval			95% HPD Credible Interval	
Isolate	Dose	Lower	Upper	Dose	Lower	Upper
K96243	2.51E+01	1.00E+01	5.99E+01	3.59E+02	1.73E+02	8.24E+02
MSHR305	5.98E+00	3.00E+00	1.23E+01	8.59E+02	6.06E+02	1.48E+03
MSHR668	4.42E+00	1.48E+00	1.06E+01	3.59E+02	2.17E+02	5.94E+02
406e	2.79E+00	1.10E+00	7.07E+00	1.89E+02	1.09E+02	3.23E+02
1026b	4.10E+00	1.65E+00	9.76E+00	1.29E+02	6.74E+01	2.52E+02
1106a	4.27E+03	2.97E+03	6.38E+03	1.00E+04	6.74E+03	1.70E+04
MSHR5855	3.50E-01	9.00E-02	9.90E-01	3.94E+01	1.15E+01	1.47E+02
MSHR5948	4.66E+00	2.01E+00	1.00E+01	2.97E+02	1.30E+02	7.08E+02
MSHR5858	4.97E+00	2.04E+00	1.24E+01	9.11E+02	5.11E+02	1.45E+03
HBPUB10134a	9.90E-01	1.00E-01	3.71E+00	4.32E+01	2.12E+01	8.62E+01
HBPUB10303a	7.40E+00	3.40E+00	1.40E+01	5.29E+01	2.40E+01	1.20E+02

The LD_50_ of HBPUB10134a was approximately 43 times lower in BALB/c as compared to C57BL/6 mice. Similarly, the LD_50_ of MSHR5855 was approximately 113 times lower in BALB/c as compared to C57BL/6 mice. The 1106a isolate, however, was calculated to have an LD_50_ approximately two times lower in BALB/c mice as compared to that calculated for C57BL/6 mice. This disparity between bacterial isolates was unexpected. When these observations were analyzed, it was determined that the differences between LD_50_ estimation for BALB/c mice and those for C57BL/6 mice were all statistically significant (*P* < 0.0001 for all strains except 1106a; *P* = 0.02) **(Table 2)**. However, when we compared the order of magnitude of the difference between the LD_50_ values calculated between the mouse strains, 1106a was significantly different from all strains (*P* < 0.008) with the exception being HBPUB10303a as the order of magnitude change was determined to not be statistically distinct from that of 1106a (*P* = 0.052). When taken together, these data further support that 1106a is an unusual clinical isolate with potentially unique attributes associated with virulence in mice.

**Table 2:** Statistical comparisons of susceptibilities of mouse strains to each *B*. *pseudomallei* clinical isolate.

Isolate of *B*. *pseudomallei*	Ratio of C57BL/6 LD50:BALB/c LD50	Comparing LD50 in BALB/c with C57BL/6	Comparing difference of LD50 in other strains with difference of 1106a
K96243	14	*P* < 0.0001	*P* = 0.0077
MSHR305	144	*P* < 0.0001	*P* < 0.0001
MSHR668	81	*P* < 0.0001	*P* < 0.0001
406e	68	*P* < 0.0001	*P* < 0.0001
1026b	31	*P* < 0.0001	*P* = 0.0001
1106a	2	*P* = 0.0224	N/A
MSHR5855	113	*P* < 0.0001	*P* < 0.0001
MSHR5948	64	*P* < 0.0001	*P* < 0.0001
MSHR5858	183	*P* < 0.0001	*P* < 0.0001
HBPUB10134a	44	*P* < 0.0001	*P* = 0.0004
HBPUB10303a	7	*P* < 0.0001	*P* = 0.0520

### Bacterial burden determined after exposure to aerosolized bacteria

**Fig 2:**
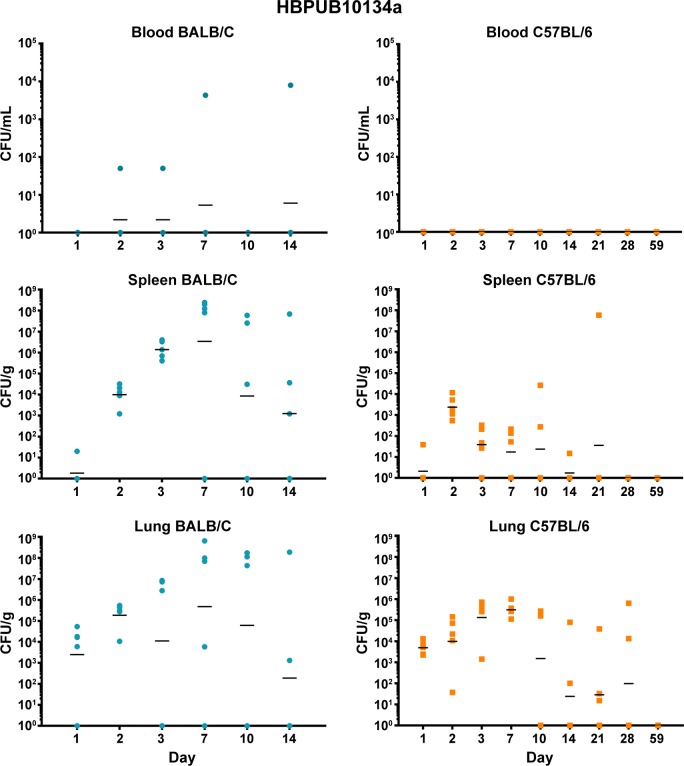
Bacterial burden observed during a serial sampling study of mice exposed to aerosolized *B*. *pseudomallei* HBPUB10134a. BALB/c mice are depicted in the top row **(blue)** and C57BL/6 mice are depicted in the bottom row **(orange)**. The CFU determinations for blood, Spleen, and lung are shown and the geometric mean is depicted with a horizontal bar.

**Fig 3:**
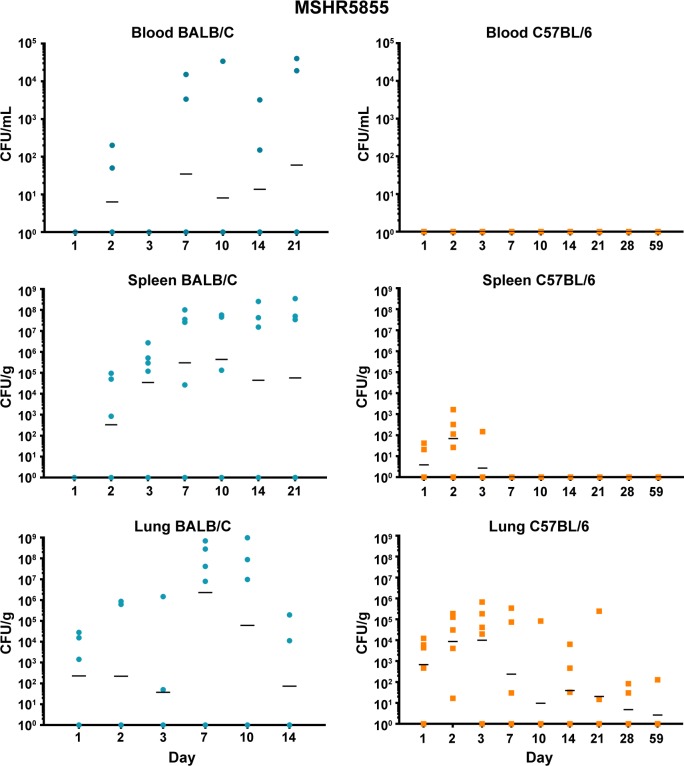
Bacterial burden observed during a serial sampling study of mice exposed to aerosolized *B*. *pseudomallei* MSHR5855. BALB/c mice are depicted in the top row **(blue)** and C57BL/6 mice are depicted in the bottom row **(orange)**. The CFU determinations for blood, Spleen, and lung are shown and the geometric mean is depicted with a horizontal bar.

**Fig 4:**
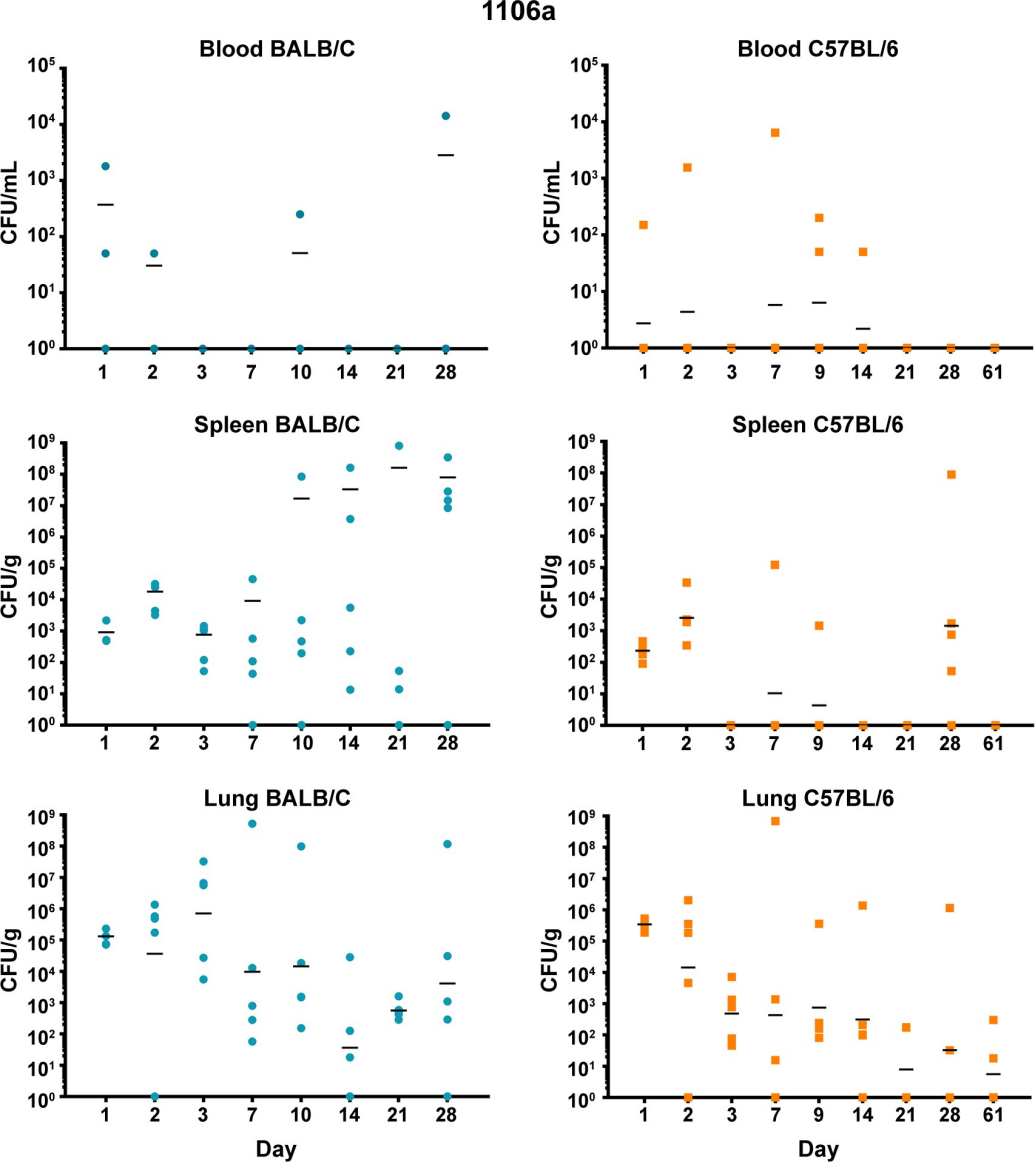
Bacterial burden observed during a serial sampling study of mice exposed to aerosolized *B*. *pseudomallei* 1106a. BALB/c mice are depicted in the top row **(blue)** and C57BL/6 mice are depicted in the bottom row **(orange)**. The CFU determinations for blood, Spleen, and lung are shown and the geometric mean is depicted with a horizontal bar.

Both BALB/c and C57BL/6 mice were exposed to low doses of aerosolized bacteria **(Table 3)** and then were euthanized and serially sampled. Due to the disparity between LD_50_ values of our most virulent strains and 1106a, the goal was to deliver bacteria based upon LD_50_ equivalents. Under these conditions, BALB/c mice succumbed to the infection by HBPUB10134a and MSHR5855 after 14 to 21 days, while after aerosol exposure to 1106a they survived until at least day 28. In contrast, C57BL/6 mice after exposure to the same strains, survived until the end of the study. The bacterial burden data demonstrated patterns that were similar when comparing HBPUB10134a and MSHR5855 **(Figs 2 and 3)**. In general, BALB/c mice were observed to have larger bacterial burdens in the spleens and lungs than in these organs in C57BL/6 mice. BALB/c mice exhibited low levels of bacteremia while C57BL/6 mice were not determined to be bacteremic (or at least were below the assay limit of detection of 50 CFU/ml). The dissemination patterns observed for 1106a were to some extent different **(Fig 4)**. This isolate was delivered at a substantially higher CFU dose as compared to that of HBPUB10134a or MSHR5855 to account for LD_50_ differences **(Table 3)**. 1106a was the only isolate that caused bacteremia in both BALB/c and C57BL/6 mice. BALB/c and C57BL/6 mice also had an appreciably higher spleen burden after exposure to 1106a after 21–28 days than after exposure after the same period to the more virulent strains. The lung burden however remained fairly similar between the BALB/c and C57BL/6 mice exposed to 1106a **(Fig 4)**.

**Table 3:** Delivered dose and LD_50_ equivalent of each *B*. *pseudomallei* isolate.

	BALB/c		C57BL/6	
Isolate	CFU	LD_50_	CFU	LD_50_
HBPUB10134a	6.0 +/- 1.4	6.1	10.9 +/- 1.3	0.2
MSHR5855	1.2 +/- 0.6	3.4	13.0 +/- 3.9	0.3
1106a	1,496.3 +/- 163.7	0.4	1,496.3 +/- 163.7	0.2

### Histopathological observations after exposure to aerosolized bacteria

**Fig 5:**
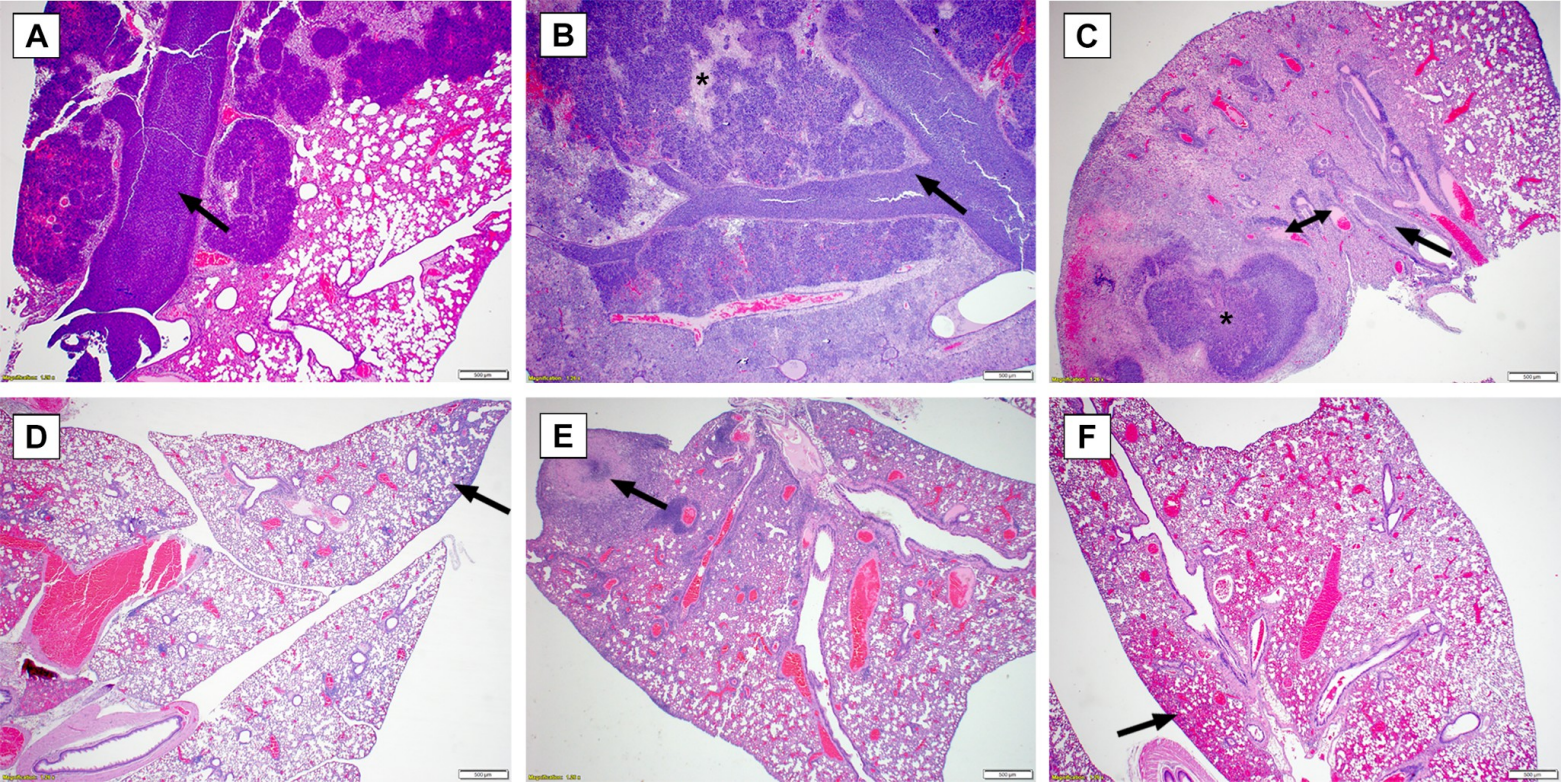
Representative Lung Pathology in mice after exposure to aerosolized *B*. *pseudomallei*. **(A)** BALB/c mouse exposed to 1106a (Day 9). The bronchus, bronchioles and surrounding alveoli are distended by suppurative inflammation and macrophages (arrow), with variable amount of edema fluid. **(B)** BALB/c mouse exposed to HBPUB10134a (Day 10). The bronchus, bronchioles and surrounding alveoli are severely distended or replaced by abundant suppurative inflammation with fewer macrophages (arrow). Edema fluid and hemorrhage also variably expand the alveoli and to a lesser extent conducting airways. Pulmonary architecture is replaced by necrotic debris in some areas (asterisk). **(C)** BALB/c mouse exposed to MSHR5855 (Day 10). The bronchus, bronchioles and surrounding alveoli are distended by suppurative inflammation and macrophages (arrow). There is marked edema fluid within the alveoli. A pyogranuloma is present with adjacent areas of necrosis and hemorrhage (asterisk). Several vessels are occluded by fibrin thrombi (double arrow). There is moderate BALT hyperplasia. The less affected area has expanded interstitium by neutrophils and macrophages. **(D)** C57BL/6 mouse exposed to 1106a (Day 9). There is minimal to mild interstitial expansion by neutrophils and macrophages (arrow); and mild BALT hyperplasia. **(E)** C57BL/6 mouse exposed to HBPUB10134a (Day 10). The interstitium is moderately expanded by neutrophils and macrophages. A focal area of necrosis replaces pulmonary architecture and resembles a poorly formed pyogranuloma (arrow). There is moderate BALT hyperplasia. **(F)** C57BL/6 mouse exposed to MSHR5855 (Day 10). There is mild expansion of the interstitium by neutrophils and macrophages. There are extensive areas of hemorrhage expanding alveoli (arrow). There are small foci (not pictured) of aggregated macrophages with few MNGCs.

**Fig 6:**
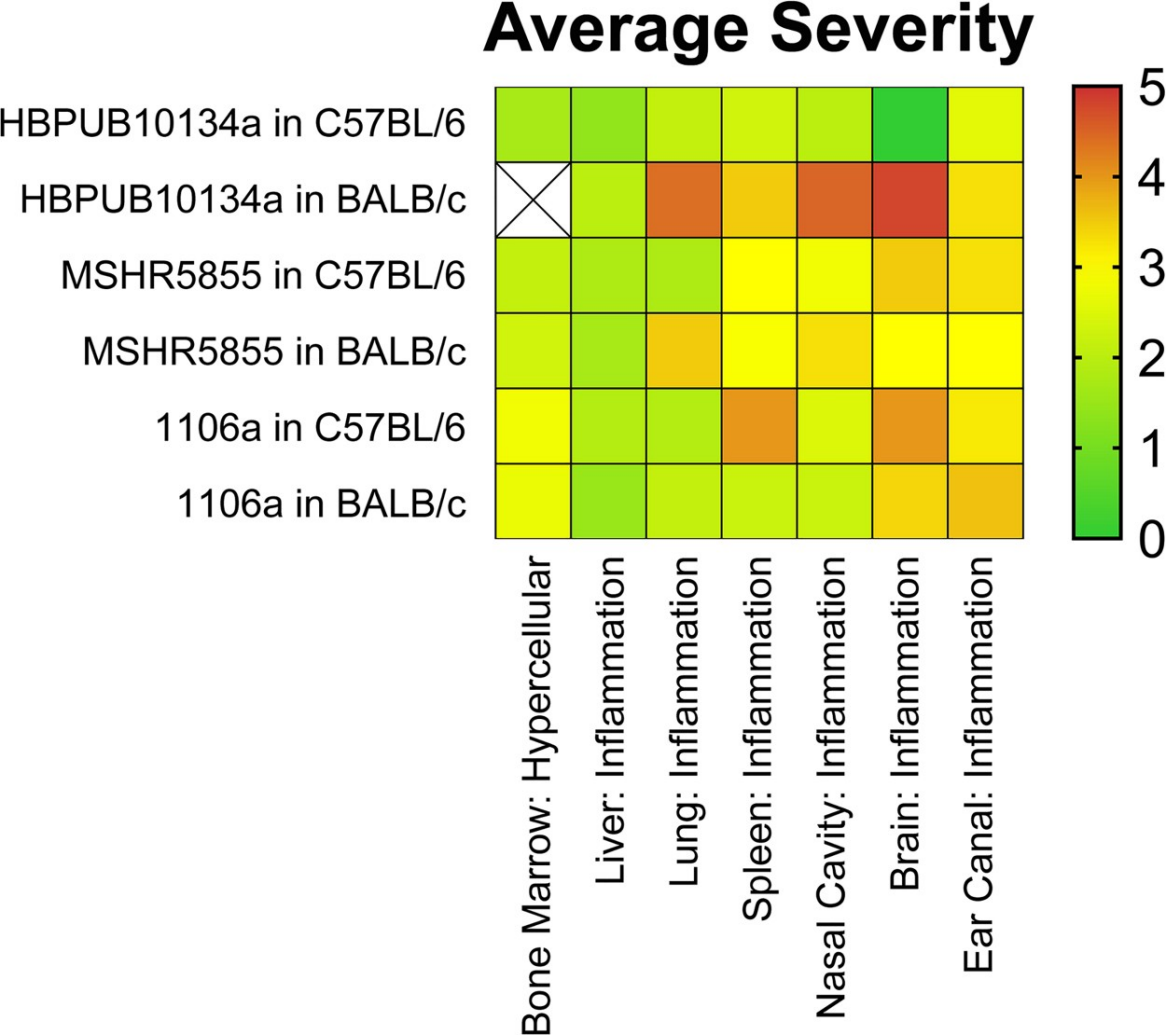
Heat map depicting the average severity observed in BALB/c and C57BL/6 mice after exposure to aerosolized *B*. *pseudomallei*.

Mice were euthanized at described time points and processed for histopathological analyses. **Fig 5** depicts common lung pathology associated with each bacterial isolate and each strain of mouse. As shown in **Fig 5** and described below, the lung was the most commonly impacted organ after exposure to aerosolized bacteria. Additionally, the histopathological scores of different organ systems were averaged and are depicted in **Fig 6**.

### *B*. *pseudomallei* HBPUB10134a: Comparative pathology in C57BL/6 and BALB/c mice

The group of C57BL/6 female mice exposed to aerosolized *B*. *pseudomallei* isolate HBPUB1034a exhibited a mild immunologic response in the airways. While observed as early as day 2 post-exposure to aerosolized bacteria, the lesions were relatively slow to develop, more consistently minimal to mild, and generally developed discrete pyogranulomas as chronic infections. Some form of lung pathology was noted in 21/34 C57BL/6 mice but only 8 of these examples exhibited moderate or greater severity. Lesions of the nasal cavity, trachea, middle ear, and olfactory lobe of the brain were rare to nonexistent among these mice, but these findings may be dependent upon the delivered dose of bacteria. Interstitial pneumonia occurred by day 10 in 13/34 mice. The liver was the only other consistently affected organ besides the lung and lesions also occurred by the second or third day in most (19/34) C57BL/6 mice. The liver pathology did not progress in severity in general; rather it was consistently low grade multifocal chronic-active inflammation. The random nature of other affected organs made establishing general conclusions more challenging. Of the animals evaluated, only two had widespread organ involvement to include the lungs, liver, spleen, and bone; and the lesions in these two cases were overall more severe. There was little evidence of lymphoid depletion, none of lymphocytolysis, and a reduced frequency of regenerative bone marrow in the C57BL/6 mice examined. The necrosuppurative bone marrow lesions which regionally spread to affect the surround soft tissue and/or spinal cord were unique to the chronic cases, as this was not observed among mice receiving a higher dose of bacteria resulting in a more acute disease course.

Overall, the lesions in the BALB/c reflected a more acute and severe disease progression. Lesions occurred more consistently, earlier on, and with greater intensity than compared to the C57BL/6 mice. Within all studies, the lung was the most commonly affected organ, with early lesions progressing from bronchiocentric suppurative pneumonia to florid bronchopneumonia, often with areas of interstitial inflammation and vascular fibrin thrombi. Well-formed pyogranulomas were a frequent finding in the more chronic cases of C57BL/6, but were overall less common in the BALB/c. Pyogranulomas were rare in the lung, but more common in the spleen in the BALB/c mice. Upper airway lesions, otitis, and encephalitis were more common in the BALB/c than C57BL/6 mice, but this may be dose dependent. The suppurative to pyogranulomatous airway inflammation was similar between the studies, but occurred with greater severity in BALB/c mice.

Both the liver and spleen were equally affected in frequency in the BALB/c mice, with the earliest lesions observed on day 2. The splenic lesions were more severe than C57BL/6 mice, while the liver lesions were fairly consistent throughout, composed of multifocal mild to moderate chronic-active inflammation and sometimes hepatocellular necrosis. Splenic lesions more readily form pyogranulomas, which were multifocal but often coalesced to encompass large areas of the spleen. There was often marked splenic hematopoiesis in the BALB/c mice, which was noted only infrequently outside of the bone marrow in the C57BL/6 mice. The extramedullary hematopoiesis maybe due to bacteremia. LPS-TLR4 interaction induces hematopoietic stem cell proliferation in the short term, while sustained LPS stimulation reduces long term proliferation [24].

Lymphoid depletion was common in the thymus of BALB/c mice (8/17); a finding rarely observed in the C57BL/6 study (3/33) but one that occurred more frequently if the mice are exposed to a higher dose of bacteria. Necrosuppurative bone lesions which affected the surrounding soft tissue and were often apparent as gross swellings of the limbs or tail were present in the more chronic C57BL/6 cases. This finding was not readily observed in any BALB/c mice; however, the limbs were not collected for histopathologic examination for that study, as no gross lesions were reported. Pulmonary hemorrhage was another feature commonly seen in the BALB/c mice (and C57BL/6 mice if exposed to a higher dose of bacteria) but was not readily observed in the C57BL/6 mice receiving approximately 6 CFU. Multinucleated giant cells (MNGCs) were not a major feature in any of the HBPUB10134a groups. MNGCs become more prevalent as lesions increase in duration and severity; however, they are considered rare in comparison to other inflammatory cell types.

### *B*. *pseudomallei* MSHR5855: Comparative pathology in C57BL/6 and BALB/c mice

In general, the histological findings associated with the MSHR5855 isolate were similar to those described above for HBPUB10134a. BALB/c mice overall exhibited higher incidence and greater severity of inflammatory lesions than C57BL/6 mice, although there was a disparity in the lung. The lung is more often affected in C57BL/6 (30/34) versus BALB/c (14/33); however, the BALB/c mice developed inflammation in greater severity with higher incidence of vasculitis and fibrin thrombi. Also, eight BALB/c mice developed well-formed pyogranulomas while none were found in C57BL/6 mice. Liver lesions were more common in BALB/c mice (29/33) versus C57BL/6 mice (10/34). Abscesses, vasculitis, and fibrin thrombi occurred in BALB/c but were not detected in C57BL/6 mice. Overall the severity of inflammation was similar between strains of mice and was generally mild. The spleen was commonly affected in BALB/c mice and pyogranulomas were prominent (14/33) while the spleen was rarely affected in C57BL/6 mice. Other lesions recorded were similar between the two strains. There were 11 recorded inflamed lymph nodes in BALB/c mice, with no similar finding identified in the C57BL/6 lymph nodes. MNGCs were not a major feature in any of the MSHR5855 groups, but occurred in greater prevalence than with HBPUB10134a infections. MNGCs became more prevalent as lesions increase in duration and severity; however, they were overall considered rare in comparison to other inflammatory cell types.

### *B*. *pseudomallei* 1106a: Comparative pathology in C57BL/6 and BALB/c mice

At the 1,500 CFU dose of 1106a, 27/29 BALB/c mice had inflammation within the nasal cavity compared to 16/31 C57BL/6 mice. The character and severity of inflammation in the nasal cavity was similar in both mouse strains; typically neutrophilic and evenly distributed between minimal to moderate severity. Only one mouse of each strain demonstrated severe inflammation. Epithelial necrosis of the nasal cavity was present in 12/29 BALB/c versus only 2/21 C57BL/6 mice. Inflammation was also the main finding in the lung for both mouse strains. The character of inflammation was variable but predominantly neutrophilic. Nearly all mice examined had at least some degree of lung pathology (29/29 BALB/c and 29/31 C57BL/6 mice). Severity of lung inflammation was almost identical between the two strains of mice, with BALB/c mice exhibiting slightly higher incidence of mild lesions (19/29 in BALB/c compared to 16/31 in C57BL/6) and 2/29 BALB/c mice with severe lesions which are not present in the C57BL/6. Other lung lesions identified rarely in both groups were necrosis, thrombus formation, edema and lymphoid hyperplasia. The inflammation affected both the alveolar lumen and interstitium in both strains, with alveolar inflammation predominating in animals euthanized early versus interstitial inflammation predominating in more chronic stages of infection. Hepatic inflammation was the predominant liver lesion in both mouse strains, affecting 14/31 C57BL/6 and 19/29 BALB/c mice. Inflammatory lesions were predominantly minimal to mild and neutrophilic in character. Mononuclear infiltrates were minimally present in both strains and considered a background finding. Hepatic necrosis was present and associated with inflammation in similar numbers of either strain of mouse (11/31 C57BL/6 and 9/29 BALB/c mice). One of the most appreciable differences in pathology observed with 1106a was the impact on the spleen. Splenic lesions were uncommon in C57BL/6 mice (1/31), but 12/29 BALB/c mice exhibited inflammation, necrosis or both.

Brain lesions were uncommon in both strains of mice with just one C57BL/6 mouse exhibiting inflammation and necrosis versus 5/29 BALB/c mice. Ear lesions were similarly common in both strains at the delivered dose (13/31 C57BL/6 and 11/29 BALB/c) and always included neutrophilic inflammation of the middle and/or inner ear. Other lesions variably present were epithelial hyperplasia, epithelial necrosis, and serocellular crusts on the tympanic membrane.

Of the examined lymph nodes, only the mandibular lymph node in BALB/c mice consistently had lesions (15/29). Most commonly this consisted of draining inflammatory cells, likely from upper airway inflammation; however, necrosis was present in 3/29 mice and lymphoid hyperplasia in 6/29. Lymphoid depletion/thymic atrophy was present in 6/29 C57BL/6 and 9/23 BALB/c mice. Lymphocytolysis was present in 5/29 C57BL/6 mice but not identified in any BALB/c mice. While a notable regenerative myeloid response in the bone marrow was present in 10/31 C57BL/6 mice and 19/29 BALB/c mice.

### Immune response after exposure to aerosolized *B*. *pseudomallei*

#### Antibody response

**Fig 7:**
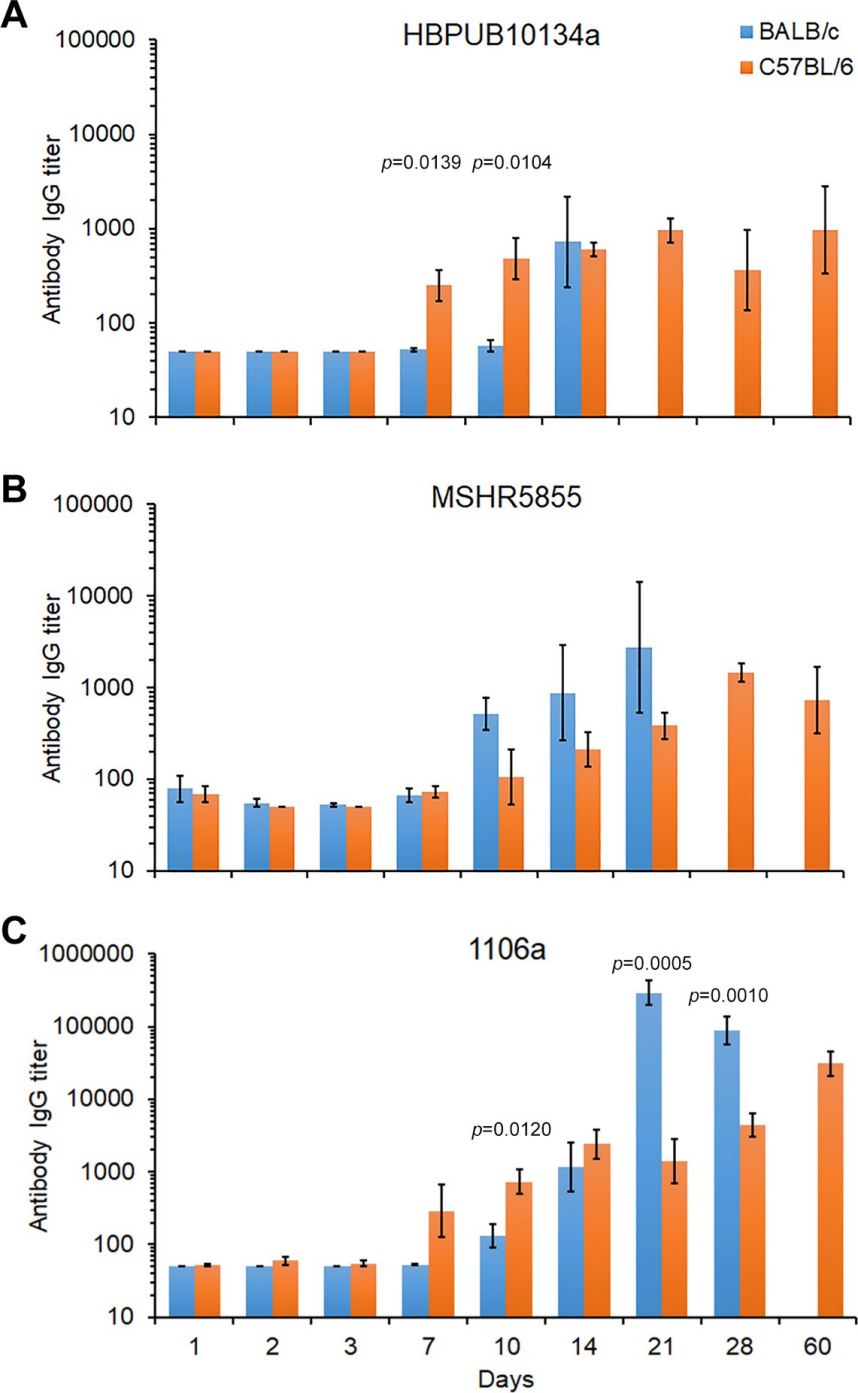
The IgG antibody response in BALB/c or C57BL/6 mice after aerosol exposure to *B*. *pseudomallei* **(A)** HBPUB10134a, **(B)** MSHR5855, or **(C)** 1106a. Blood was drawn from mice (N = 5 for each time point) after exposure to *B*. *pseudomallei* strains when possible after 1, 2, 3, 7, 10, 14, 21, 28, and 60 days. ELISA was performed on the sera at least once in triplicate for each group of BALB/c **(blue bars)** and C57BL/6 **(orange bars)** mice and IgG titer reported as the geometric means with the standard error of the mean depicted. Due to their susceptibility not enough BALB/c mice survived to end of study, to determine the antibody levels, whereas C57BL/6 exposed mice survived until the end of the study (60 days). Significant differences in the IgG levels between BALB/c and C57BL/6 mice at specific time points after exposure is shown above the results.

Sera were collected to evaluate the antibody response generated against killed bacteria as the antigen. As illustrated in **Fig 7**, the antibody response against killed *B*. *pseudomallei* K96243 was distinguishable between the three strains of bacteria after exposure.

***B*. *pseudomallei* HBPUB10134a.** When we compared the antibody response in BALB/c and C57BL/6 mice to HBPUB10134a **(Fig 7A)**, we first detected an IgG response at 7 days post-infection that occurred in C57BL/6 mice but not in BALB/c for the same period (difference *P* < 0.050). Similarly, at 10 post-infection the antibody response to HBPUB10134a in C57BL/6 mice continued to rise but not in BALB/c mice (*P* < 0.050). It was not until 14 days post-infection there was a noticeable IgG response in BALB/c mice while the IgG titer slowly increased in C57BL/6 mice at the same time. Twenty-one days after exposure to HBPUB10134a there were no BALB/c survivors, while in the C57BL/6 mice survivors the antibody titer slowly increased and generally remained the same until the end of the study at 60 days.

***B*. *pseudomallei* MSHR5855.** When we examined the IgG antibody response after exposure to MSHR5855, unlike in mice exposed to HBPUB10134a, we observed a slight increase in the IgG titer in both BALB/c and C57BL/6 mice at 7 days **(Fig 7B)**. The IgG response in BALB/c mice increased from 10 days to 21 days post-infection, after which time no BALB/c mice survived. In contrast, the IgG response in C57BL/6 mice slowly increased from day 10 to 28 days after infection and decreased in survivors at 60 days. There was only a significant difference in the IgG response in C57BL/6 mice exposed to MSHR5855 or HBPUB10134a at 7 days (*P* = 0.0268) but not in BALB/c mice in the same studies.

***B*. *pseudomallei* 1106a.** The antibody response to the least virulent *B*. *pseudomallei* strain examined was different in both mouse strains than the IgG response to the two previous highly virulent *B*. *pseudomallei* strains examined **(Fig 7C)**. In infected BALB/c mice we first noted an increase in the IgG titer at 10 days post-infection, and it continued to increase up to 21 days and decreased at 28 days post-infection. There was a modest but significant difference in the IgG titer at 21 days after infection between BALB/c mice exposed to MSHR5855 or 1106a (*P* = 0.0452). In exposed C57BL/6 mice we saw the IgG titer increased at 7 days compared to that in BALB/c mice for the same period. Thereafter, we identified a moderate increase in the IgG titer in C57BL/6 mice to 60 days. Over the course of this study there were significant differences in the antibody titer between BALB/c and C57BL/6 mice at 10 days post-infection (*P* = 0.0120), 21 days (*P* = 0.0005), and 28 days (*P* = 0.0010). We also noted differences between the IgG response in C57BL/6 mice exposed to MSHR5855 and 1106a after 10 days (*P* = 0.0464), 14 days (*P* = 0.0048), 28 days (*P* = 0.0405), and 60 days (*P* = 0.0088). There were also some differences in the antibody response between C57BL/6 exposed to HBPUB10134a and 1106a 14 days (*P* = 0.0390) and 60 days post-infection (*P* = 0.0277). Likewise, we only noted significant differences in the IgG titer in BALB/c mice exposed to MSHR5855 or 1106a after 21 days (*P* = 0.0452). Overall, we observed higher IgG titers in both BALB/c mice and C57BL/6 mice exposed to 1106a than in mice exposed to the other two highly virulent *B*. *pseudomallei* strains.

#### Cytokine/Chemokine expression

**Fig 8:**
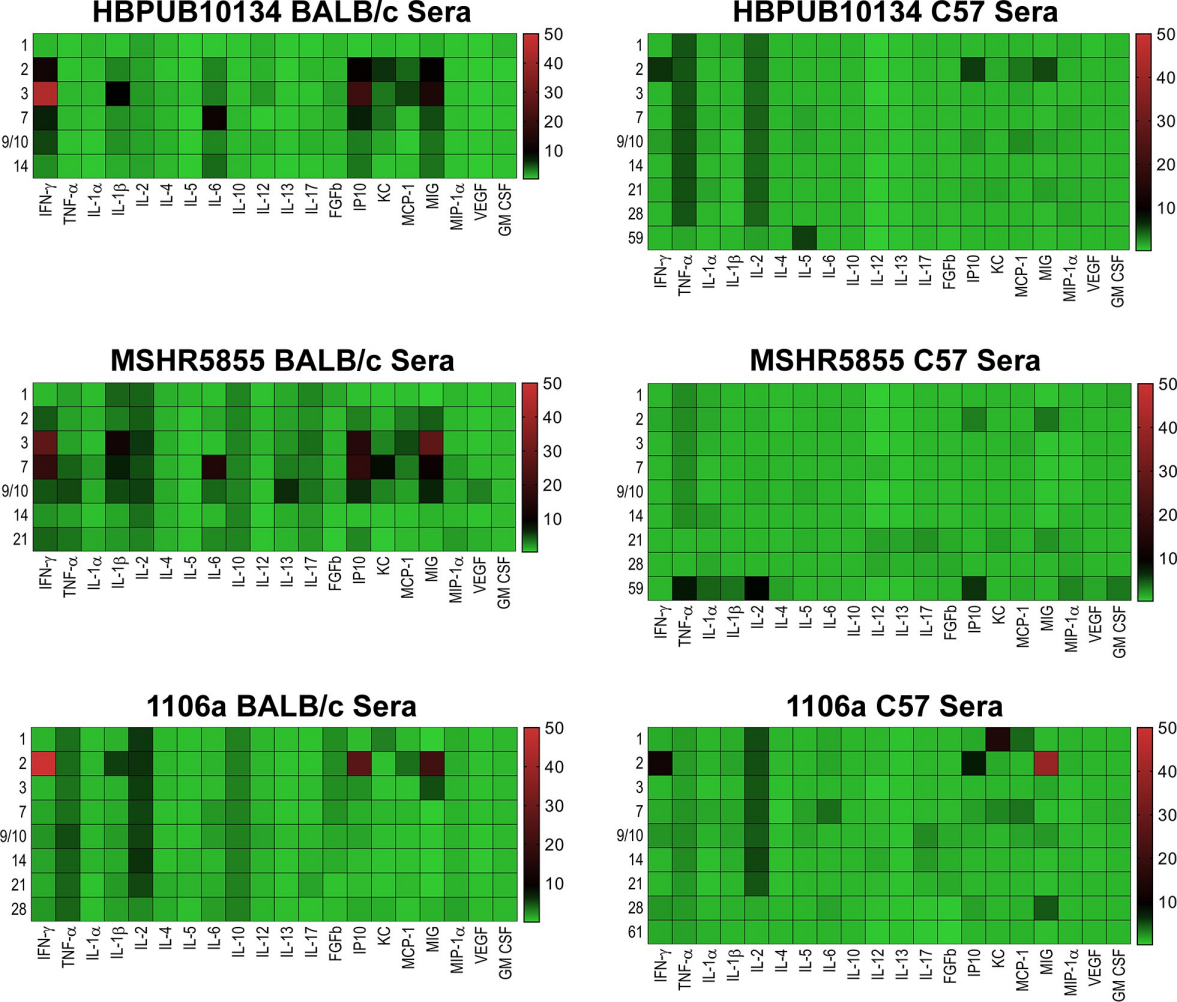
Changes in cytokine/chemokine levels in sera from BALB/c and C57BL/6 mice after aerosol exposure to *B*. *pseudomallei*. Sera were examined for cytokine/chemokine expression at different times after exposure of BALB/c or C57BL/6 mice to HBPUB10134a, or MSHR5855, or 1106a. For each time point after exposure N = 5, and geometric means were determined. Fold changes in cytokines/chemokines in sera were determined by dividing the amount in geometric means (pg/ml) determined after exposure at a specific time by the amount found in sera from naïve, normal BALB/c or C57BL/6 mice (pg/ml). For cytokines/chemokines in sera from naïve BALB/c mice, N = 6, and from C57BL/6 mice, N = 5. The number on the left of each panel shows the days after exposure. The bar on the right of each heat map shows the fold-change in expression of each cytokine/chemokine after time of exposure.

**Fig 9:**
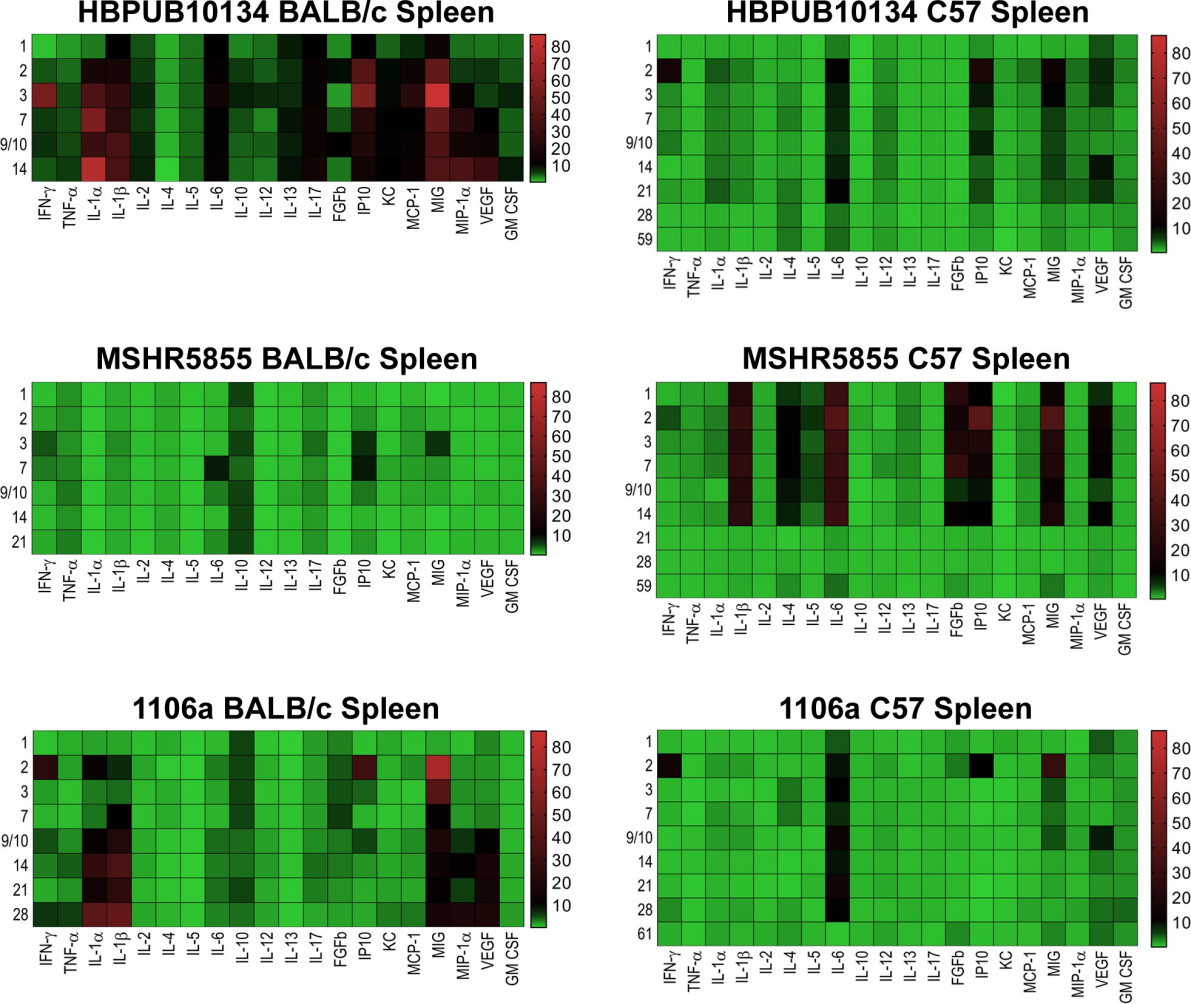
Changes in cytokine/chemokine levels in spleen homogenates from BALB/c and C57BL/6 mice after aerosol exposure to *B*. *pseudomallei*. Spleen homogenates were examined for cytokine/chemokine expression at different times after exposure of BALB/c or C57BL/6 mice to HBPUB10134a, or MSHR5855, or 1106a. For each time point after exposure N = 5, and geometric means were determined. Fold changes in cytokines/chemokines in spleen homogenates were determined by dividing the amount in geometric means (pg/ml) determined after exposure at a specific time by the amount found in lung homogenates from naïve, normal BALB/c or C57BL/6 mice (pg/ml). For cytokines/chemokines in spleens from naïve BALB/c mice, N = 4 and for C57BL/6 mice, N = 5. The numbers on the left of each panel shows the days after exposure. The bar on the right of each heat map shows the fold-change in expression of each cytokine/chemokine after time of exposure.

**Fig 10:**
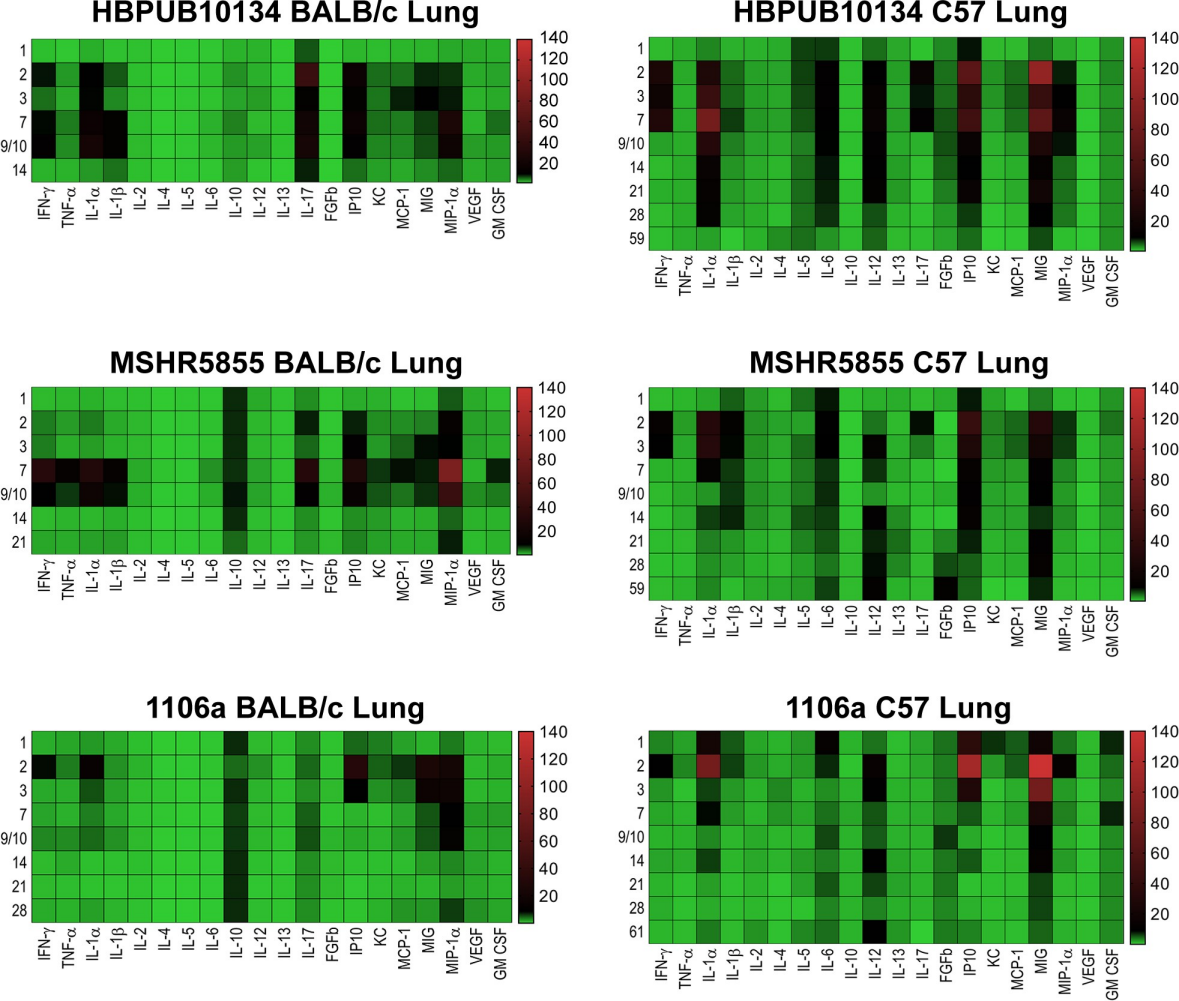
Changes in cytokine/chemokine levels in lung homogenates from BALB/c and C57BL/6 mice after aerosol exposure to *B*. *pseudomallei*. Lung homogenates were examined for cytokine/chemokine expression at different times after exposure of BALB/c or C57BL/6 mice to HBPUB10134a, or MSHR5855, or 1106a. For each time point after exposure N = 5, and geometric means were determined. Fold changes in cytokines/chemokines in sera were determined by dividing the amount in geometric means (pg/ml) determined after exposure at a specific time by the amount found in spleen extracts from naïve, normal BALB/c or C57BL/6 mice (pg/ml). For cytokines/chemokines in lungs from naïve BALB/c mice, N = 5, and for C57BL/6 mice, N = 5. The numbers on the left of each panel shows the days after exposure. The bar on the right of each heat map shows the fold-change in expression of each cytokine/chemokine after time of exposure.

Cytokine/chemokine analyses were performed on all samples collected with a goal of comparing the changes in expression between the three strains of *B*. *pseudomallei* in three different tissues/sera in two strains of mice. **Figs 8–10** illustrate the fold- increase calculated for each of these cytokines/chemokines at different times after exposure. **S1 Table** reports individual cytokine/chemokine concentrations, and statistical analyses of changes compared to naïve mice in BALB/c and C57BL/6 mice.

***B*. *pseudomallei* HBPUB10134a.** This isolate was the most virulent strain from Thailand in our *B*. *pseudomallei* strain panel and initiated overall the greatest expression of cytokines/chemokines after infection. In sera, the BALB/c mice exhibited a notable increase in expression of IFN-γ, IP10, and MIG starting at day 2–3 post-infection **(Fig 8A)**. In sera of C57BL/6 mice (despite no culture evidence of bacteremia) we saw upregulated expression levels of the same cytokines on day 2 although not as high as seen in BALB/c mice, but these quickly tapered off by day 3 **(Fig 8B)**. However, sera collected from C57BL/6 mice revealed sustained modest increases of both TNF-αand IL-2 that were not seen in sera from exposed BALB/c mice. Spleen samples collected from BALB/c mice exposed to HBPUB10134a showed the largest number of cytokines/chemokines expressed beginning on day 2 through day 14 after exposure **(Fig 9A)**, that generally corresponded with the bacterial burden observed in these samples **(Fig 2A)**. With the exception of IL-4, the level of expression of all the other cytokines/chemokines detected were significantly (*P* = 0.0317-< 0.0001) increased at one point during the course of the study, for example IFN-γ, IL-1α, IL-1β, IL-6, IP10, and MIG. Spleens collected from C57BL/6 mice infected with this strain showed fewer cytokines/chemokines expressed early and showed a sustained increased expression of IL-6, IP10, and MIG with an early (2 days post-infection) transient increase of IFN-γ **(Fig 9B)** When cytokines/chemokines expressed in lungs of C57BL/6 mice exposed to HBPUB10134a were examined **(Fig 10B)**, there was clearly an earlier and more intense immune response than in lungs of BALB/c mice exposed to HBPUB10134a **(Fig 10A)**. We observed significant increased expression in lungs of C57BL/6 of IFN-γ, IL-1α, IL-12, IL-17, IP-10, MIG, and MIP-1α but with variable magnitudes **(Fig 10B)**. At the same time, there were fewer and generally lower amounts of expression of cytokines/chemokines in lungs of BALB/c mice than in lungs of C57BL/6 mice, such as IFN-ℽ, IL-1α, IL-1β, IL-17, IP-10, MIG, and MIP-1α.

***B*. *pseudomallei* MSHR5855.** The MSHR5855 clinical isolate was the most virulent Australian isolate in our strain panel in both BALB/c and C57BL/6 mice, and it was for the most part similar in virulence as the HBPUB10134a isolate. We found significant levels of a few cytokines/chemokines in sera of BALB/c mice exposed to MSHR5855, although a very low dose of bacteria was delivered (approximately 1 CFU) **(Fig 8C)**. The type of cytokines/chemokines found in sera from BALB/c mice was similar to that found in sera from HBPUB10134a infected BALB/c mice **(Fig 8A and 8C)**. Three to seven days after exposure, we detected the highest levels of IFN-ℽ, IL-1β, IL-6, IP-10, and MIG in sera from exposed BALB/c mice. In contrast, in sera from C57BL/6 mice exposed to MSHR5855, which did not exhibit appreciable bacteremia, we saw only a modest but significant increase in TNF-α, IL-2 and IP-10 but not until day 59 post-exposure. We noted, however, there were several important differences in the immune response in mice exposed to HBPUB10134a or MSHR5855 in spleen and lung samples **(Figs 9 and 10)**. In spleens from MSHR5855 infected C57BL/6 mice, the number and amount of cytokines/chemokines detected in spleen homogenate from C57BL/6 mice were significantly elevated over that found in spleens from MSHR5855 infected from BALB/c mice **(Fig 9C and 9D)**. Those amounts of cytokines/chemokines found significantly elevated (*P*<0.0001) in spleens of C57BL/6 mice were IL-1β, IL-4, IL-5, FGF-β, IP-10, MIG, and VEGF. These findings were unexpected, given the disparity of the bacterial burden observed in the spleens in the different strains of mice. It was also interesting that virtually all of the increased expression of cytokine/chemokines in C57BL/6 spleen homogenates occurred primarily up to day 14 post-exposure.

When lung homogenates from BALB/c mice were examined, MSHR5855 induced highest levels of IFN-ℽ, TNF-α, IL-1α, IL-1β, IL-17, and MCP-1 between 3–10 days post-exposure **(Fig 10C)**. A modest but significant and sustained levels of IL-10 expression was seen, as well as a more robust expression of MIP-1α response in BALB/c mice was observed **(Fig 10C)**. The lungs collected from C57BL/6 mice exposed to MSHR5855 showed similar cytokines/chemokines expressed in lungs from HBPUB10134a infected lungs **(Fig 10B and 10D)**. Over the course of the study, expression of significant levels (*P* < 0.0001) of IFN-γ, IL-1α, IL-1β, IL-6, IL-12, IL-17, IP-10, and MIG were observed **(Fig 10D)**.

***B*. *pseudomallei* 1106a.** The 1106a isolate offered a unique opportunity to characterize melioidosis in the two strains of mice with similar calculated LD_50_ values. We challenged each cohort of mice with the same number of CFU. However, it was important to note that because of the attenuation of 1106a, our challenge target was much larger (i.e. approximately 1,500 CFU for 1106a and ≤ 13 CFU for the other bacterial isolates). Thus, it was impossible to completely rule out that some of these observations noted were due to increased CFU being introduced into the lungs and not just solely based on differences in bacterial isolates. Not surprisingly, the cytokine expression profiles were similar in either BALB/c or C57BL/6 mice after exposure in sera **(Fig 8E and 8F)**. We detected significant levels of IFN-γ, IP-10, and MIG after 1–2 days after exposure, with higher levels seen in sera from BALB/c mice, except for MIG which was higher in sera from C57BL/6 mice. When we examined cytokines/chemokines present in spleen homogenates prepared from 1106a infected mice, the total number and level of cytokines/chemokines were intermediate between HBPUB10134 and MSHR5855 **(Fig 9A, 9C and 9D)**. We found in spleens from BALB/c mice 2 days after infection significant amounts of IFN-γ, IL-1α, IL-1β, IL-10, IP-10, and MIG **(Fig 9E)**. At the same time, these same cytokines/chemokines were expressed either at lower levels in spleens from 1106a infected C57BL/6 mice or were absent **(Fig 9F)**. Seven to 28 days after infection, significant amounts of IL-1α, IL-1β, MIG, and VEGF were expressed in spleens from BALB/c mice. Over this same period, we found only significant amounts of IL-6 expressed in spleens from infected C57BL/6 mice. When we examined cytokines/chemokine expressed in lungs of 1106a infected mice, generally the cytokines/chemokines expressed in lungs of 1106a infected BALB/c mice were similar to that seen in lungs of BALB/c mice infected with HBPUB10134a or MSHR5855, although we observed a few differences. For example, in BALB/c exposed to 1106a we noted a sustained expression of IL-10 in lungs of mice exposed to both MSHR5855 and 1106a, but it was barely detected in BALB/c lungs after exposure to HBPUB10134a. Also, there was generally a higher expression of IL-17 in lungs of BALB/c mice exposed to HBPUB10134a than was seen in lungs of MSHR5855 or 1106a exposed mice. When we compared the expression of cytokines/chemokines in BALB/c or C57BL/6 1106a exposed mice, we detected little to no IL-6, IL-12, FGF-β or GM-CSF in lungs of BALB/c mice, but we found sometimes significant amounts (*P* < 0.0001) in 1106a infected lungs of C57BL/6 mice. Furthermore, we noted the early (2–3 days post-exposure) expression of significantly higher amounts (*P* < 0.0001) of IL-1α, IP-10, and MIG in lungs of C57BL/6 mice compared to the expression levels recorded in lungs from BALB/c mice. On the other hand, we observed overall more MIP-1α expressed in lungs of BALB/c than in lungs of C57BL/6 mice.

### Cellular changes during infection with *B*. *pseudomallei* MSHR5855

**Fig 11:**
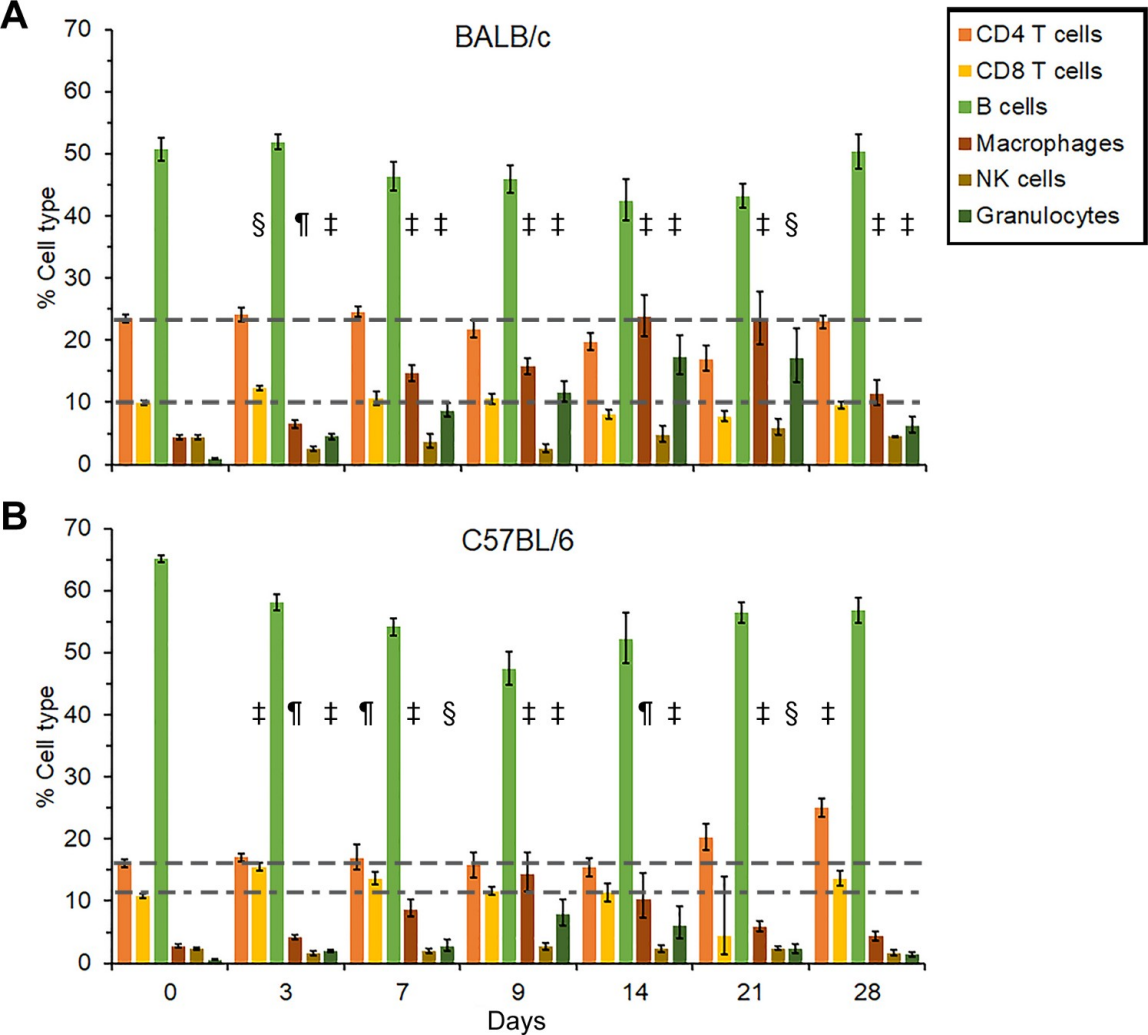
Cellular changes in spleens collected from serially sampled mice. BALB/c **(A)** and C57BL/6 mice **(B)** were exposed to *B*. *pseudomallei* 1106a. In most cases N = 5.

**BALB/c mice**. In splenocytes from BALB/c mice infected with *B*. *pseudomallei* MSHR5855 there was an early (3–7 days post-infection) increase in both CD4+ and CD8+ T cells (*P* < 0.001-*P* < 0.0001) as well as in monocyte/macrophages, NK cells, and granulocytes **(Fig 11)**. Ten days after infection, there was a decrease in the CD4+ and CD8+ T cells population, while the inflammatory cell population remained high (*P* < 0.001–0.0001) up to 14 to 21 days post-infection **(Fig 11A)**. At day 21, the number of NK cells were back to normal, but at the same time the number of monocyte/macrophages and granulocytes remained high. All BALB/c mice had succumbed to infection or were euthanized by day 28.

**C57BL/6 mice.** In contrast, in the splenocyte population from C57BL/6 mice there was an early influx of CD4+ and CD8+ T cells 3 days post-infection, and their levels remained high (*P* < 0.0001) through 28 days of the study **(Fig 11B)**. There was a moderate increase in the number of monocyte/macrophages and granulocytes starting at day 3 (*P* < 0.010-*P* < 0.0001) throughout the study (28 days post-infection), but they started to decrease after 14 days after exposure. Overall, the amount of inflammatory cells was not as high as seen in BALB/c mice for the same period. Hence, we noted a pronounced early T cell response in BALB/c mice that was temporary, but in C57BL/6 mice this increase was sustained throughout the study. In contrast, the trends observed with inflammatory cells was reversed in BALB/c versus C57BL/6 mice.

### Cellular changes during infection with *B*. *pseudomallei* 1106a

**Fig 12:**
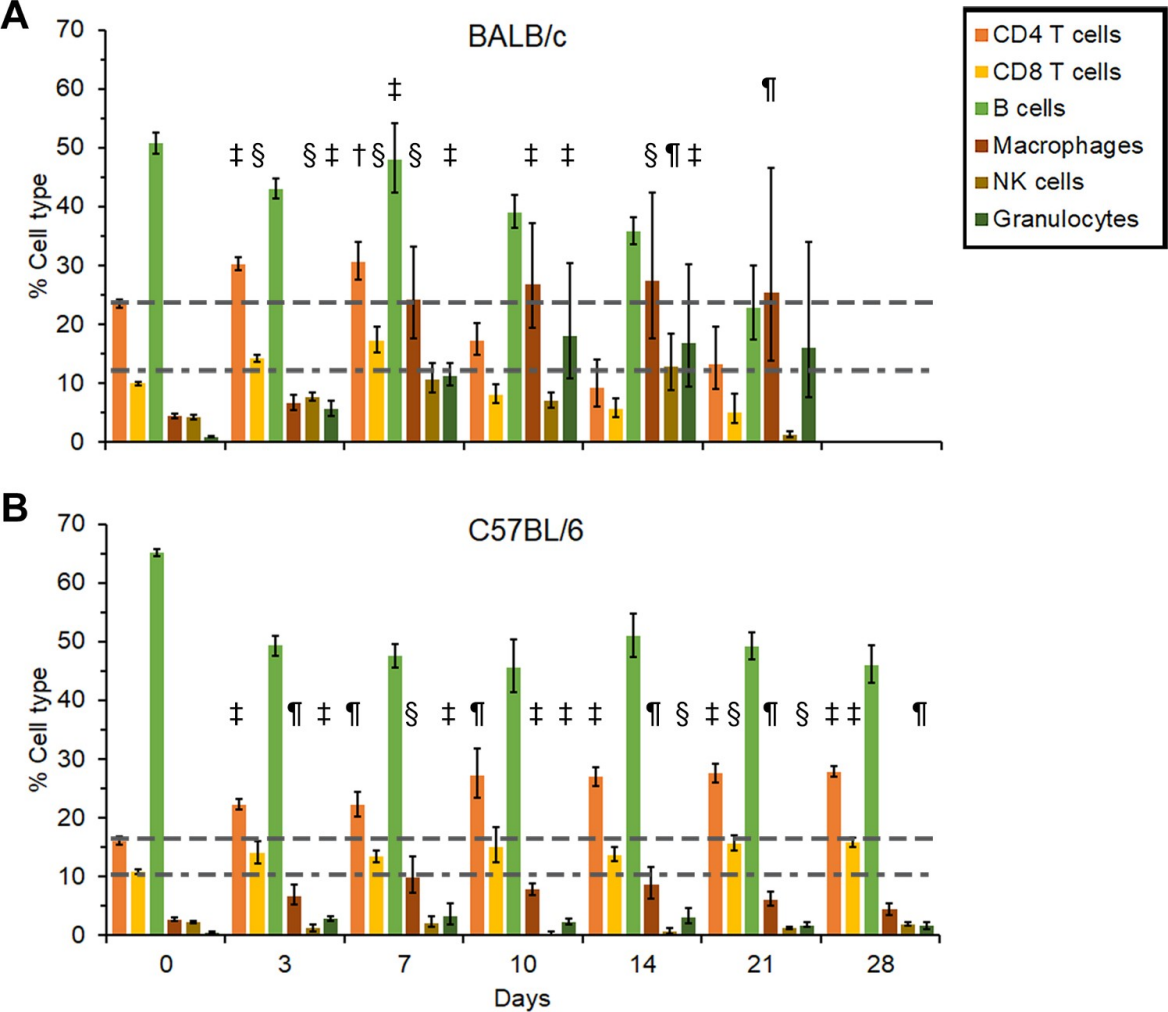
Cellular changes in spleens collected from serially sampled mice. BALB/c **(A)** and C57BL/6 mice **(B)** were exposed to *B*. *pseudomallei* MSHR5855. In most cases N = 5.

**BALB/c mice**. We examined the changes in the cellular composition of the splenocyte population after infection with *B*. *pseudomallei* 1106a **(Fig 12)**. There was a slight increase in the CD4+ and CD8+ T cell population early (3 days post-infection) after infection but essentially normal amounts up to 28 days post-infection in BALB/c mice **(Fig 12A)**. However, there was a gradual but significant increase in inflammatory cells, such as monocyte/macrophages, and granulocytes after 3 days post-infection (*P* < 0.01-< 0.0001). The increase in these inflammatory cells reached a maximum between 14 to 21 days before we observed a decrease in the monocyte/macrophage and granulocyte population 28 days post-infection.

**C57BL/6 mice**. We noted an increase in CD8+ T cells (*P* < 0.0001) early (3–7 days, post-infection) after infection with *B*. *pseudomallei* 1106a, and we saw a significant increase in CD4+ T cells (*P* < 0.0001) later (21 to 28 days post-infection) after infection **(Fig 12B)**. At the same time, we detected a moderate increase in monocyte/macrophage and granulocyte cells that started 3 days post-infection (*P* < 0.01) which peaked after 9 days (P < 0.0001) and slowly decreased after 28 days post-infection. Thus, there was a significant increase in inflammatory cells (monocyte/macrophages and granulocytes) in BALB/c mice early after infection with *B*. *pseudomallei* 1106a, but a significant CD4+ T cell response to the pathogen later in C57BL/6 mice.

## Discussion

The work reported here focused on characterizing the mouse model of inhalational melioidosis using the BARDA/DTRA strain panel. Importantly, we have down-selected to four strains for further work. As we have previously reported, we have chosen to continue to use K96243 (a low passage and defined sample) in order to bridge data in the literature [18]. Additionally, we will continue to use HBPUB10134a and MSHR5855 because these represent the most virulent clinical isolates from Thailand and Australia, respectively. Lastly we will continue to study 1106a because it is the least virulent clinical isolate in the panel and is unique as measured by several parameters.

As demonstrated in this report and previous work, the HBPUB10134a and MSHR5855 isolates are reasonably similar in many respects. This is generally true for LD_50_ analyses, bacterial burden observed, and histopathological analyses. *B*. *pseudomallei* 1106a; however, is distinguishable in several aspects. This clinical isolate has the highest LD_50_ in the intraperitoneal model of infection [17]. It was also observed that the day 21 and Day 60 LD_50_ calculation in the intraperitoneal model for 1106a did not change significantly. At day 21 the calculated intraperitoneal LD_50_ was 40,000 CFU and at day 60 the LD_50_ was 41,000 CFU. Most of the other strains had substantially lower LD_50_s when calculated at day 60 as compared to day 21, demonstrating that even seemingly attenuated strains could still produce lethal infections with an extended observation period. This was not the case with 1106a. Again, in this current report, 1106a had the highest LD_50_ value (regardless of mouse strain used) of any of the strains tested. 1106a was the only strain that was observed to cause bacteremia in C57BL/6 mice **(Fig 4)** and based upon histopathological analyses demonstrated appreciably altered pathogenesis (i.e. lack of pyogranuloma formation compared to the more virulent isolates). Lastly, it is worth noting that the difference in LD_50_ values calculated for BALB/c and C57BL/6 mice for 1106a were significantly different, but the order of magnitude of this variation was markedly different than nearly all of the other strains examined **(Table 3)**. In our hands, 1106a appears to act similarly regardless of whether we used BALB/c or C57BL/6 mice. One hypothesis is that this strain could be less impacted by the differential immune response generated in C57BL/6 compared to that in BALB/c mice. We have previously reported that 1106a is one of the most cytotoxic strains in vitro and it does elicit distinct immune responses in vivo [17, 25]. Thus, this strain could have a higher propensity to bypass an acute infection-state and result in a more “chronic” scenario.

The overall conclusion of the histopathology presented in this report supports these assertions. Not surprisingly, the lung (as the portal of entry for the bacteria in this model) was the most commonly affected organ across all bacterial strains, dosing, and mouse strains. HBPUB10134a caused the most severe lung lesions, followed by MSHR5855 and then 1106a. Pyogranulomas were consistently present in MSHR5855 infected BALB/c mice, but also occurred commonly in C57BL/6 mice exposed to HBPUB10134a. Pyogranuloma formation was rare in all 1106a-infected mice. In 1106a-infected mice, lung inflammation was common in both strains but overall less severe; and the later-stage mice tended to develop strictly interstitial inflammation versus outright alveolar inflammation and pyogranuloma formation in the other bacterial strains.

Spleen and liver pathology were both common, with the exception of the spleen in 1106a exposed C57BL/6 mice. Severity of liver inflammation and necrosis was generally mild across all strains; however, inflammatory cell infiltrates and/or hepatocyte necrosis occurred in high frequency as a background finding which may confound results in these studies. Severity of splenic inflammation was more variable, with BALB/c mice generally exhibiting moderate to severe lesions after the first few days post-infection and C57BL/6 mice exhibiting minimal to mild lesions from HBPUB10134a and MSHR5855 while rarely developing any lesions from 1106a infection. The higher the dose and more virulent the bacterial strain, the more likely the accumulation of inflammatory cells and necrosis of the nasal cavity spreads to the inner ear and brain. BALB/c mice were more susceptible to this and it was a rare finding in C57BL/6 mice. The presence of MNGCs has been described as the hallmark of melioidosis [26]; however, is considered overall rare in relation to other inflammatory cell types, but appears to be most prevalent in BALB/c mice infected with strain MSHR5855.

There are several important differences associated with these *B*. *pseudomallei* isolates. The two most virulent isolates HBPUB10134a and MSHR5855 are both isolates from tracheal sputum samples, whereas 1106a was collected from the pus of a liver abscess [16]. Additionally, we have previously reported differences in the LPS of these isolates [17]. The Australian isolate MSHR5855 has a LPS banding pattern with a higher range of molecular weights compared to either HBPB10134a or 1106a clinical isolates. We must also reiterate that due to the disparate LD_50_ values, the mice exposed to 1106a are challenged with a significantly higher number of CFU (i.e. 1,500 CFU for 1106a compared to 6 CFU for HBPUB10134a). While this scenario affords the unique opportunity to challenge BALB/c and C57BL/6 mice at comparable doses, the number of bacteria used to achieve LD_50_ equivalents may account for some of the differences observed in vivo (i.e. bacteremia associated with 1106a).

Overall, the cytokine and chemokine trends reflect higher levels of inflammation in BALB/c mice than in C57BL/6 mice during infection with 1106a, particularly at later time points. The enhanced tolerance of C57BL/6 mice to *B*. *pseudomallei* infection when compared with infection in BALB/c mice is consistent with other published studies [27–29]. These observations suggest that specific host pathways are determining factors in host mortality.

The C57BL/6 and BALB/c mouse strains have historically been polarized towards a Th1- and Th2-dominant immune responses, respectively. Our cytokine profiles resulting from the *B*. *pseudomallei* infections have thus illustrated how the chronic C57BL/6 model compares to the acute BALB/c, uncovering critical cytokine secretion patterns that support the Th1/Th2 paradigm. Monokine induced by IFN*-*γ (MIG) and IFN-γ-inducible protein 10 (IP-10) are Th1 cell–recruiting chemokines that promote the migration of activated T-cells to affected tissues [30–34]. We observed large increases in MIG and IP-10 in the lungs and spleens of C57BL/6 and BALB/c mice infected with HBPUB10134a or MSHR5855 that remained high throughout the course of infection. However, in C57BL/6 mice, IP-10 induction was faster, starting on day 1 post-infection in the lungs, and both, MIG and IP-10, persisted longer, beyond 28 days. In contrast to C57BL/6 mice, the levels for both chemokines in the lungs of BALB/c mice decreased after 10 days post-infection. Mice infected with 1106a exhibited a similar pattern of MIG and IP-10 production with rapid and more intense levels in the lungs of C57BL/6 relative to BALB/c mice. The presence of bacteremia correlated with higher levels of IFN-γ, MIG and IP-10 in serum in both strains of mice.

Both MIG and IP-10 are thought to be primarily induced by IFN-γ [31]. The production of IFN-γ in the lungs, the site of entry, was greater in C57BL/6 mice than BALB/c mice within the first 3 days post-challenge with HBPUB10134a or MSHR5855. In BALB/c mice, IFN-γ production appeared in the lungs 7 days post-challenge and became more pronounced at later time points. This IFN-γ spike was consistent with infections with other *B*. *pseudomallei* strains in both mice and humans [35–38]. The earlier and more potent IFN-γ response in the lungs of C57BL/6 mice may have potentiated the earlier, more prolonged and robust MIG and IP-10 expression relative to BALB/c mice [39]. Given the established involvement of MIG and IP-10 in mononuclear cell and T-cell migration, it is likely that the differences in MIG and IP-10 expression in BALB/c and C57BL/6 mice affected recruitment of activated mononuclear and T-cells to affected organs in the host [31, 40, 41]. Since MIG and IP-10 were among the most highly upregulated chemokines in the lungs of C57BL/6 mice, which were able to more effectively control *B*. *pseudomallei* infection, it is possible that both chemokines were important in promoting early clearance of bacteria in addition to any roles they may have later during infection in the spleens of BALB/c mice. It is also feasible that differences in MIG, MIP-1α, and IP-10 expression, between the two strains of mice, may reflect or cause detrimental dysregulation of the T-cell mediated response during later time points of *B*. *pseudomallei* infection.

MIP-1α is a proinflammatory chemokine that acts as a lymphocyte attractant, primarily recruiting CD8+ T lymphocytes and B cells, and mitigates hematopoietic stem cell proliferation [42, 43]. Of note, in the absence of earlier IFN-γ induction there was a prolonged increase in MIP-1α in the lunges of BALB/c mice [39]. Unlike, MIG and IP-10, which have limited chemo-attractive activity to neutrophils, MIP-1α serves as their potent recruiter [44–46]. In C57BL/6 mice, 2 to 3 days post-infection, there is an increase in MIP-1α which rapidly recruits neutrophils to the site of infection, and once the initial control of *B*. *pseudomallei* is achieved, the levels of MIP-1α recede. In BALB/c mice, MIP-1α secretion is prolonged due to the inability to control the infection, resulting in increased neutrophil recruitment, subsequently exacerbates tissue damage. Similar to the effects of MIG, the differences in MIP-1α likely imitate the involvement of a T-cell-mediated response that differs in the infections between the BALB/c and C57BL/6 mice. The differences in MIG in these mouse models have been previously observed by our group and others during infection by multiple routes with different strains of *B*. *pseudomallei* [18, 25]. Re-stimulation of lung derived C57BL/6 leukocytes by the gram-negative bacillus resulted in increased production of IL-12, which promotes naïve T cell differentiation into Th1 cells while suppressing Th2 development [47–49]. Furthermore, there was no detectable IL-10 production, a potent Th2 stimulating and Th1 inhibiting cytokine, in the lungs of C57BL/6 while a strong response was detected in BALB/c mice infected with MSHR5855 or 1106a. Previously, it was described that increased IL-12 production by C57BL/6 macrophages resulted in increased IFN-γ and the synthesis of effector molecules for bacterial killing, such as lysosomal enzymes (β-glucuronidase) and nitric oxide, while similar responses in BALB/c macrophages were absent leading to failed bacterial clearance [50]. This earlier containment of the *B*. *pseudomallei* infection at the site of primary infection may partially explain the lack of observed bacteremia in C57BL/6 relative to BALB/c mice challenged with MSHR5855 or HBPUB10134a. Furthermore, the bacterial burden in the lungs of C57BL/6 mice starts to drop or at least stabilizes by day 3 post-infection with MSHR5855 or HBPUB10134a while a reduction in BALB/c mice is only observed 7 days post-infection. The continuous increase in macrophage and granulocyte populations in spleen extracts of BALB/c mice infected with MSHR5855, may have been due to more *B*. *pseudomallei* dissemination from the lungs resulting in greater splenic bacterial burden and the inflammatory cells functioning more like “Trojan horses” rather than bactericidal effector cells. A similar pattern was also observed in BALB/c mice infected with 1106a; however, by day 28 post-infection the percent of the inflammatory cells was receding with the mice transitioning from an acute to a more chronic state of infection. In contrast, C57BL/6 mice were able to control the infection in the lungs and significantly reduced the bacterial dissemination to the spleen. Also, the zenith of the macrophage and granulocyte population in the spleens of C57BL/6 mice was much lower than in BALB/c mice, peaking at 7 days post-infection, even though there were no detectable CFUs by that time point.

A strong IL-6 response was present in the lungs and spleens of C57BL/6 mice challenged with all three isolates, but absent in all BALB/c counterparts aside from mice challenged with HBPUB10134a. IL-6 is known to induce the differentiation of CD8+ T cells into cytotoxic T cells, enhance immunoglobulin synthesis by activated B cells, as well as promote naïve CD4+ T cells transition into Th17 cells, while inhibiting Treg differentiation [51]. One may postulate that elevated levels of IL-6 in the lungs of mice challenged with MSHR5855 or HBPUB10134a would promote Th17 cell differentiation and production of IL-17 in C57BL/6 while increased IL-10 levels in BALB/c mice would inhibit IL-17 levels [52–54]. Surprisingly, higher levels of IL-17 are observed in BALB/c mice infected with MSHR5855 or HBPUB10134a than in C57BL/6. Alternatively, a majority of the IL-17 production during the early stages of infection in the lungs may be driven by the IL-17+ γδ T cells while Th17 cells appear at later stages of infection, specifically in BALB/c mice [55]. Since Th17 cells play a critical role in bacterial clearance and require TGF-β in concert with IL-6 for differentiation, it is possible that *B*. *pseudomallei* is able to alter TGF-β or other critical factors necessary for optimal Th17 response in BALB/c mice [56–59]. Elevated levels of IL-4, IL-5, and IL-6 in the lungs and spleen of C57BL/6 relative to BALB/c mice challenged with MSHR5855 or 1106a may have contributed to increased proliferation and differentiation of B cells resulting in a higher percentage of the total splenocytes, although, total antibody titers were higher in BALB/c mice [60–62]. Antibody titers were higher in both strains of mice challenged with 1106a relative to MSHR5855 or HBPUB10134a, which may partially explain the 1106a attenuated virulence.

A protective role of activated neutrophils that are rapidly recruited to the lungs after intranasal *B*. *pseudomallei* infection in C57BL/6 mice was previously described [63]. Prolonged and elevated levels of IL-6 in the lungs of C57BL/6 mice may initially serve to recruit and protect neutrophils and minimize their depletion from the site of infection [64], after which apoptosis induced shedding of IL-6R from neutrophil surface and the formation of IL-6/sIL-6R complex leads to IL-6 trans-signaling. This results in the recruitment of mononuclear phagocytes and the phagocytosis of the neutrophil infiltrate, which in turn favors the resolution of inflammation [65]. Although, excessive recruitment of mononuclear-cells may transition from an acute to a chronic inflammatory state [66].

Another pro-inflammatory cytokine, IL-12, which induces the production of IFN-γ and favors the differentiation of T helper 1 cells was highly elevated in the lungs of C57BL/6 infected mice but absent in BALB/c [67]. Increased survival after lethal challenge was observed in BALB/c mice vaccinated with non-viable *Burkholderia mallei* that was fortified with IL-12 relative to the formulation deficient in the cytokine [68]. Similarly, we observed higher levels of IL-1β in spleens of infected BALB/c mice throughout the course of infection. IL-1β expression is required for control of *B*. *pseudomallei* infection in the lungs. One mechanism that triggers IL-1β expression is inflammasome activation. *B*. *pseudomallei* activates the NLRP3 and NLRC4 inflammasomes, which causes expression and secretion of IL-1β and IL-18 and leads to restriction of *B*. *pseudomallei* infection in the lungs [69]. The augmented expression of IL-1β in spleens of BALB/c mice may reflect an effort by the host to control disseminated bacteria in the spleen. Given that both C57BL/6 and BALB/c mice express functional NLRP3 and NLRC4 inflammasomes, it is unlikely that a host genetic defect is responsible for difference in IL-1β expression in the spleens of these animals during *B*. *pseudomallei* infection. An emerging paradigm suggests that other mechanisms for IL-1β cleavage and activation exist outside of the inflammasomes. Alternative proteases to caspase-1 that may be involved in such mechanisms have not yet been identified, but it is possible that some of the IL-1β detected in the spleens of *B*. *pseudomallei* infected animals may also be cleaved by such an alternative mechanism.

IL-1α was also differentially expressed between the BALB/c and C57BL/6 mice, with higher fold-change in the spleens of BALB/c mice, but higher fold-change on days one and two in the lungs of C57BL/6 mice. IL-1α is a proinflammatory cytokine that is either surface-expressed or secreted. Activation of NF-kB signaling pathways is associated with surface display of IL-1α, and secretion of IL-1α occurs upon inflammasome activation and cleavage by caspase-1 [70]. Thus, the early expression of IL-1α, particularly in the lungs of C57BL/6 mice on day 2 after infection, likely represents secreted IL-1α as a result of inflammasome activation. IL-1α, in turn, stimulated precursor IL-1β production by macrophages [71]. Furthermore, IL-1α expression in combination with increased IL-12, another inflammatory cytokine, likely promotes early clearance and marshalling of the adaptive immune response in C57BL/6 mice. This is in contrast to the increased IL-1α in the lungs of BALB/c mice that is accompanied by increased IL-10 expression. As an anti-inflammatory cytokine, it is possible that IL-10 mitigates some of the Th1 mediated inflammation that is required to control *B*. *pseudomallei* infection in the lungs of BALB/c mice. The increases in IL-1α observed later during infection in the spleens of BALB/c mice could indicate activation and secretion of IL-1α by inflammasome activation, though further data, such as caspase-1 activation and IL-18 expression would be required to validate this hypothesis. This may be part of an effort by the host to control bacterial loads in the spleen since inflammasome activation controls replication of *B*. *pseudomallei* [69, 72–75]. Ultimately, IL-1α expression often corresponds with neutrophil influx, which is likely involved in controlling infections with a range of *B*. *pseudomallei* strains [18, 76].

In conclusion, BALB/c mice were observed to be in a greater inflammatory state compared to C57BL/6 mice after the first few days of infection. This corresponds with previous studies using other *B*. *pseudomallei* strains in these mouse models. These studies also documented differences in the expression of inflammatory cytokines and chemokines such as MIG, IP-10, KC, and IL-1 in BALB/c and C57BL/6 mice [18, 25, 77]. Future investigations could employ knockout mice to assess the requirement for key cytokines and chemokines and their direct effects on *B*. *pseudomallei* infection. Aside from the Th1- and Th2-biased immune responses in C57BL/6 and BALB/c, respectively, other factors to consider involve differences in antibody compositions and complement opsonizing activity between the two inbred strains [78]. In the BALB/c mice the more diverse innate IgA repertoire might bind to *B*. *pseudomallei* without neutralizing the pathogen while a more potent T regulatory cell response would enable for greater suppression of cell mediated bactericidal effector responses [78–80]. Prior work demonstrated the kinetics of deposition of C3b in C57BL/6 is faster than in BALB/c mice [81]. This would impact both arms of the immune response and partially influence the efficacy of antibody responses, phagocytosis efficiency, and immune cell recruitment and activation in these two mouse strains [82–84]. Furthermore, gender dimorphism, in relation to terminal complement pathway, is evidenced by reduced complement activity in female relative to male mice in both inbred strains [85]. Future work can circumvent these limitations with the addition of outbred strains, such as CD-1, for comparison of immunological responses and identification of correlates of protection.

This study adds to the growing body of literature describing inhalational melioidosis in mouse models of disease. The analyses described here are meant to discern and characterize similarities and differences in bacterial isolates of varying disease potentials and geographic locations of origin. As has been described previously and is understood by the *B*. *pseudomallei* research community, this bacterium results in an extremely heterogeneous infection and can be challenging to characterize. However, our data presented here will help to further understand this disease process and allow for better standardization of animal models and more robust testing of novel medical countermeasures.

## Supporting information

S1 Table Numeric data and statistical analyses for all cytokines measured in the serial sample experiment. (XLSX)
